# New insights in the composition of extracellular vesicles from pancreatic cancer cells: implications for biomarkers and functions

**DOI:** 10.1186/s12953-014-0050-5

**Published:** 2014-11-18

**Authors:** Susanne Klein-Scory, Mahnaz Moradian Tehrani, Christina Eilert-Micus, Kamila A Adamczyk, Nathalie Wojtalewicz, Martina Schnölzer, Stephan A Hahn, Wolff Schmiegel, Irmgard Schwarte-Waldhoff

**Affiliations:** IMBL, Medical Clinic Knappschaftskrankenhaus Bochum GmbH, Ruhr-University Bochum, In der Schornau 23-25, 44892 Bochum, Germany; Functional Proteome Analysis, German Cancer Research Center (DKFZ), Im Neuenheimer Feld 280, 69120 Heidelberg, Germany; Molecular Gastrointestinal Oncology MGO, Ruhr-University Bochum, Universitätsstraße 150, 44780 Bochum, Germany; Medical Department, Medical Clinic Knappschaftskrankenhaus Bochum GmbH, Ruhr-University Bochum, In der Schornau 23-25, 44892 Bochum, Germany

**Keywords:** Extracellular vesicles, Exosome, Secretome, Proteomics, Affinity purification, Biomarker, Pancreatic cancer

## Abstract

**Background:**

Pancreatic cancer development is associated with characteristic alterations like desmoplastic reaction and immune escape which are mediated by the cell-cell communication mechanism and by the microenvironment of the cells. The whole of released components are important determinants in these processes. Especially the extracellular vesicles released by pancreatic cancer cells play a role in cell communication and modulate cell growth and immune responses.

**Results:**

Here, we present the proteomic description of affinity purified extracellular vesicles from pancreatic tumour cells, compared to the secretome, defined as the whole of the proteins released by pancreatic cancer cells. The proteomic data provide comprehensive catalogues of hundreds of proteins, and the comparison reveals a special proteomic composition of pancreatic cancer cell derived extracellular vesicles. The functional analysis of the protein composition displayed that membrane proteins, glycoproteins, small GTP binding proteins and a further, heterogeneous group of proteins are enriched in vesicles, whereas proteins derived from proteasomes and ribosomes, as well as metabolic enzymes, are not components of the vesicles. Furthermore proteins playing a role in carcinogenesis and modulators of the extracellular matrix (ECM) or cell-cell interactions are components of affinity purified extracellular vesicles.

**Conclusion:**

The data deepen the knowledge of extracellular vesicle composition by hundreds of proteins that have not been previously described as vesicle components released by pancreatic cancer cells. Extracellular vesicles derived from pancreatic cancer cells show common proteins shared with other vesicles as well as cell type specific proteins indicating biomarker candidates and suggesting functional roles in cancer cell stroma interactions.

**Electronic supplementary material:**

The online version of this article (doi:10.1186/s12953-014-0050-5) contains supplementary material, which is available to authorized users.

## Background

Pancreatic cancer is the fifth most common cancer-related cause of death in Europe [1] and one of the most aggressive forms of human cancer. The diagnoses occur only at late stages when the patients have developed metastases, and the five-year survival rate of patients is only 5%. Up until now it has not been possible to decide whether the metastatic ability is a characteristic of the pancreatic cancer cell, or whether this is due to the delayed diagnosis. Pancreatic cancer often develops after or during pancreatic inflammation, and the differential diagnosis is complex [2]. There is an urgent need to find early and specific pancreatic cancer biomarkers, and it is necessary to develop further strategies for the treatment of this fatal disease; thus far, no curative therapies are available. Therefore, each biomarker discovery approach for pancreatic cancer should also pay attention to therapeutic implications and functional roles.

The development of pancreatic cancer is accompanied by some characteristic events: the ability to escape immune responses, the attraction of stroma cells in the desmoplastic reaction, the intravasative capacity of pancreatic cancer cells, and the low vascularization of pancreatic tumours, as well as the frequently found thromboses [3]. These characteristic processes are mediated by mechanisms of intercellular communication and by the microenvironment of the cells.

To deepen the understanding of these processes, our group and others have used the conditioned medium of cancer cells. This conditioned medium, or secretome, of cancer cells contains the whole of the released proteins of the cells, including regularly and aberrantly processed proteins. Consequently, the entirety of the released proteins is considered to be a discovery platform for the identification of tumour biomarker candidates [4]. There are different mechanisms to release proteins from cells, such as classical secretion and exocytosis, shedding by proteolytic activities, unspecific release by apoptosis, and the release of small vesicles [5]. The extracellular microvesicles (EVs) are released from most normal, diseased and neoplastic cells, and EVs are found in different body fluids, like plasma, ascites fluid, urine and saliva, and used to identify biomarkers of diseases [6]. However, in these approaches, the identity and origin of the vesicles are usually not exactly clarified, and EVs are prepared in different ways [7].

Over the last few years, EVs have increasingly become the focus of different research studies, and the current knowledge about EVs is summarized in some recent reviews [8-10]. Different EVs should play a prominent role in cell communication performing manifold functions. They facilitate invasion, modulate the mechanisms of thrombosis, can induce chemoresistance, and are mediators of immune surveillance (reviewed in [11])*.* The released vesicles mediate the ability of tumour cells to alter the environment, and help the cells during invasion or attachment to the extracellular matrix. These functions of EVs are executed by the diverse constituent molecules, and the elucidation of the composition of these vesicles is of major interest. Recent publications have shown that EVs contain specific proteins, like CD63/Lamp3, CD9 and SDCBP/Syntenin, and ribonucleic acid instrumentations [12,13]. Proteomic description of EVs of tumour cell types, especially from colon, breast, head and neck, prostate cancers and melanoma, have been published ([6] and special issue in Proteomics, 2013), but in depth information about pancreatic carcinoma cell derived EVs is not available so far. To close this gap, the present manuscript is going to describe the protein content of the EVs of pancreatic cells, and combine this information with postulated functions or assigned tasks in pancreatic carcinogenesis [14].

## Results

### Extracellular vesicles preparations obtained via ultracentrifugation are not pure vesicular samples

For the sample preparations, the conditioned media were prepared and subjected to a differential centrifugation protocol as described in the Materials and Methods section. The conditioned media were collected from serum free cultures, in order to avoid the contamination of the samples with calf serum components, such as albumins and bovine EVs. The secretome samples of the pancreatic cells contained about 5–20 μg protein per 10^6^ cells, and the crude pellet after the centrifugation procedure contained only about 0.2-0.4 μg protein per 10^6^ cells.

**Figure 1:**
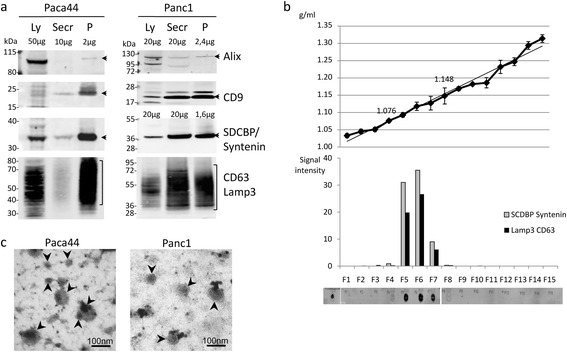
**EVs from pancreatic cancer cells: a)** **Immunoblots of different samples: proteins characteristic for EVs were enriched in pellets (P) after ultrafiltration and ultracentrifugation, compared to secretome (Secr) or lysate samples (Ly).** **b)** The crude extracellular vesicle samples in an OptiPrep gradient centrifugation. The EV markers Syntenin and CD63/Lamp3 were found in fractions with densities of 1.07-1.15 g/ml as illustrated by dot blots. **c)** Transmission electron microscope (TEM) pictures of 120,000 g pellets of Panc1 and Paca44. Lipid bilayers surrounding the vesicles are visible (arrowheads).

The characteristic proteins (compiled in [15]) for the EVs like syntenin, CD9, CD63 and Alix revealed an enrichment of EVs in these crude preparations as compared to the secretome and cell lysates (Figure 1a). Some of the EV marker proteins, such as CD9 or Syntenin, are more than twentyfold enriched in the ultracentrifugation samples when compared to the secretomes or cell lysates. Furthermore, the ultracentrifugation pellets were subjected to an OptiPrep gradient centrifugation to test whether the crude preparations of the vesicles corresponded to the typical densities of the EVs, as described earlier [16]. The fractions with the densities of 1.08-1.15 g/ml contained the EV marker proteins CD63 and syntenin (Figure 1b).

Aliquots of the crude preparations of different cells were inspected by TEM and, in fact, the pictures showed that the preparations contained vesicles, which were surrounded by a lipid bilayer membrane (Figure 1c). The vesicular content of the crude preparations was also supported by results after detergent treatment to destroy the lipid bilayers (Additional file 1: Figure S1a). Syntenin and CD9 marker proteins were distributed between the ultrafiltrate and the flow through of a 100 kDa filtration when the samples were treated with Triton ×100 or with a solubilisation buffer, but not after treatment with a low pH buffer. Additionally, the labelling with the membrane stain PKH67 or PKH26 delivered a picture of bright fluorescent particles which fit to the vesicular character of the preparations (Additional file 1: Figure S1b).

From all of these findings, we conclude that the samples produced in combination with ultracentrifugation were suitable for analysis by mass spectrometry as a crude vesicular preparation. However, the samples were not pure vesicular probes, but contained further electron dense particles. To enhance the purity of our vesicular samples, we performed the affinity purification discussed as the gold standard for isolating pure vesicles [10,17,18].

### Affinity purification is a successful procedure to enrich extracellular vesicles

Based on the proteomic analyses of the crude vesicle preparations and secretomes, we chose membrane proteins as anchors for the affinity purification with magnetic beads. We favoured the membrane protein TACSTD1/Epcam to establish affinity purification of the EVs, because it is specific for epithelial cells and it is overexpressed and retained in pancreas carcinoma cells [19] and has not been reported to be altered by mutation.

We probed the secretomes and crude EV preparations of six pancreatic ductal cell lines (Additional file 1: Figure S2a). Based on the high amount of TACSTD1/Epcam in the Paca44 samples which was also confirmed by mass spec results (21 peptides in Paca44 secretome versus 8 peptides in Panc1 secretomes), Paca44 was the most suitable source to isolate the EVs by anti-TACSTD1/Epcam coated beads. This cell line, established from a primary tumour, carries the most common genotype changes occurring during pancreatic carcinogenesis (mutated K-ras, mutated p53, deleted p16Ink4a, and wildtype smad4 genes) [20]. (The ascites derived cell line A818-4 also expressing some amount of EPCAM was disregarded for this approach, because it is not mutated in p53 and smad4 and because these cells can not easily be produced in very large numbers.) As a second cell line we selected Panc1 cells which also express some TACSTD1/Epcam anchor protein. This cell line was isolated from a primary tumour and also displays genetic alterations typically found in pancreatic cancers [20]. As expression levels of TACSTD1/Epcam were much lower in Panc1 cells as compared to Paca44 and might be limiting the efficiency of affinity purification, we additionally used the membrane protein MFGE8/lactadherin for purification of Panc1 derived EVs. MFGE8 displayed a very high number of peptides (286) in mass spectrometric analyses of crude EV samples from Panc1 (but not Paca44) cells. The immune blots confirmed that the protein MFGE8/lactadherin is overrepresented in the Panc1 secretome and EV preparations (Additional file 1: Figure S2b).

**Figure 2:**
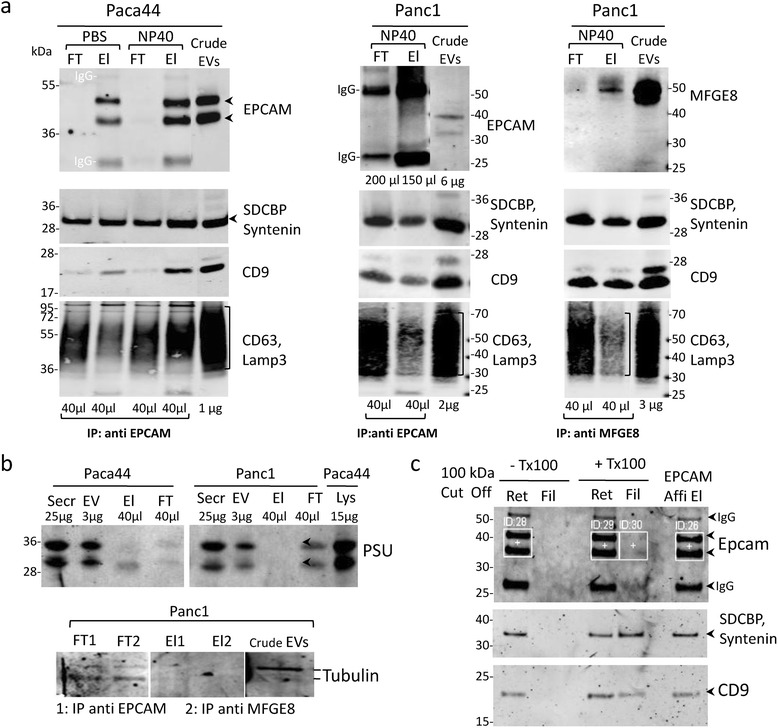
**Affinity purifications of EV samples: a)** **Using the anchor proteins of EVs TACSTD1/Epcam or MFGE8 the exosomal marker proteins as CD9, SDCBP/Syntenin and CD63 were enriched in the eluates (El) after affinity purifications of EVs whereas no or smaller amount of exosomal proteins were detected in flow through (FT).** Samples were eluted with PBS or NP40. **b)** Immunoblots confirmed that proteins like proteasome subunit alpha and tubulin beta partially remain in the flow through. **c)** Treatments of affinity purified samples with detergents (Triton × 100) allowed the passage of exosomal marker proteins through the ultrafilter membrane (cut off at 100 kDa). Ret, retentate; Fil filtrate.

To prepare the affinity pure samples, we produced the crude EV preparations and subsequently incubated them with specific bead complexes as described below. After affinity purification, the magnetic bead bound fractions (eluates), as well as the unbound fractions (flow through), were controlled by immune detection. The eluates of Paca44 derived preparations contained enriched amounts of the TACSTD1/Epcam and of the EV marker proteins (Figure 2a and Additional file 1: Figure S3). Obviously, by the immune detection, the affinity purification procedure was more efficient with the Paca44 samples than with Panc1 samples using both membrane anchors, TACSTD1/Epcam and MFGE8.

While some exosomal specific proteins were enriched within the purified samples, other protein groups, like the proteasome subunit proteins or tubulins, were detected in the flow through, only. Whereas these proteins have been described as components of the exosomes earlier [21], they in fact seem to be contaminants of the crude preparations (Figure 2b).

We wished to further proof the vesicular nature of these affinity preparations. As binding of the vesicles to the ferromagnetic beads can barely be resolved without destroying the vesicles, it was not possible to display the affinity pure samples in the electron microscope. Alternatively, we again used a detergent treatment followed by ultrafiltration (100 kDa cut off) to differentiate between protein complexes and membrane vesicles.

Treatment with triton X 100 led to a partial release of EV marker proteins syntenin and CD9 from the affinity-pure samples into the flow-through (Figure 2c). In addition, the affinity preparations were stained for membrane proteins (TACSTD1/Epcam and EGFR) and analysed by FACS (data not shown). The samples prior to and after affinity purification were labelled with fluorescence-conjugated antibodies detecting the membrane specific proteins, and with membrane stains like PKH67. The fluorescence signals were reduced in the flow through fraction of affinity purification versus the initial crude EV samples, assuming that the flow through fraction had been depleted of membrane vesicles.

In summary, the affinity purification procedure as described above appears adequate to strongly reduce contamination of protein complexes from real vesicular components.

### Proteomic characterization of the EV preparations

#### Compilation of proteomics data

The protein catalogues of the mass spectrometry results were produced from the secretomes and crude vesicle preparations of Panc1, Paca44 and HPDE cells and from affinity purified EV preparations from Panc1 and Paca44 cells. In addition, a catalogue of Panc1 whole cell lysate was available. The numbers of found proteins identified and the mass spectrometry details are compiled in Table 1. The complete protein catalogues compiling 4096 proteins in total are listed in an additional excel data file (Additional file 2) and illustrated in Venn diagrams (Additional file 1: Figure S4a,b).

**Table 1:** **Compilation of mass spectrometry analysis data**

**Cells**	**Paca44**			**Panc1**							**HPDE**	
**Sample**	**SECR**	**EVs crude**	**EPCAM affi EVs**	**lysate**	**SECR**	**EVs crude**	**EVs crude**	**EVs crude**	**EPCAM affi EVs**	**MFGE8 affi EVs**	**SECR**	**EVs crude**
**# IPIs**	1312	627	477	1184	1187	1940	749	508	415	292	1042	1343
**# GS**	1147	587	449	1470	1034	1647	688	477	384	276	911	1176
**Sum of peptides**	22965	5336	3042	19960	24431	31624	6725	3986	1914	1085	20409	19309
**Range of peptides**	1-453	1-314	1-79	1-608	1-1231	1-1962	1-420	1-358	1-46	1-66	1-940	1-1570

# IPI, number of protein accession IPI; # GS, number of gene symbols; *SECR*, secretome samples; *EVs crude*, pellet after ultrafiltration and ultracentrifugation; *EPCAM affi EVs*, affinity purified samples using anti-EPCAM beads; *MFGE8 affi EVs*, affinity purified samples using anti-MFGE8 and anti-mouse beads.

**Figure 3:**
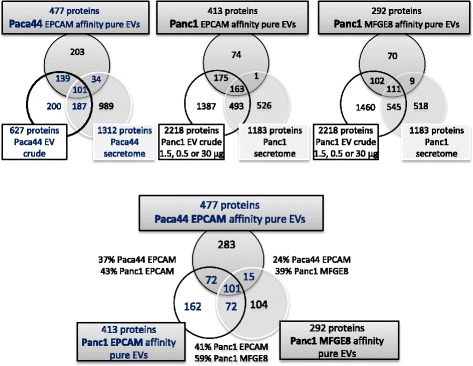
**Venn diagrams compile the comparison of the mass spectrometry results from Paca44 and Panc1.** Based on the IPI numbers proteins found in affinity pure samples of Paca44 and Panc1, were matched with proteins in crude samples and the whole secretomes of corresponding cells: 42% of the Paca44 proteins and 17% and 23% of Panc1 were exclusively measured in affinity pure samples. The number of shared proteins in affinity purification of Paca44 and Panc1 is reduced if the anchor protein is changed.

We were first interested in differences between the diverse compartments (secretome, crude vesicles, affinity-purified vesicles) within one cell line. A comparison of the protein lists from crude and affinity purified EVs and the secretome is shown in the Venn diagrams (Figure 3). Of note, just about the half of the proteins identified in the affinity-pure sample of Paca44 (477 proteins) had shown up in the crude EV sample and most of the novel protein are not found in the secretome either, suggesting that the depth of proteomic analysis of the EVs was significantly increased by affinity purification.

The affinity purification of the Panc1 samples was less effective using both anchor proteins. On the other hand, we had more comprehensive data available for crude EV preparations in this case. Consistently, the number of proteins identified in the affinity pure samples of Panc1 EVs was lower (415 proteins) and the overlap with the crude sample higher (85%) as compared to Paca44 EVs.

A similar pattern of overlaps with the secretome and the crude sample on the one hand and distinct proteins in the affinity pure sample was detected with the sample from MFGE8 affinity purification, which comprises 292 proteins in total.

Interestingly, however, both catalogues of the affinity pure samples share only 173 proteins (Additional file 1: Figure S4c) and both affinity pure samples of Panc1 gave 535 proteins. This finding may suggest, that the affinity purification procedures with these two anchor proteins enrich distinct vesicle subpopulations.

Secondly, we were interested in changes of the proteomic composition through affinity purification of vesicles common to both cell lines. Here we again present the data in the Venn diagram; further aspects are discussed later on.

The overlap between the two different Epcam affinity samples appears moderate at a first glance (37 and 43%, respectively, in Figure 3). These lists, however, were to be produced from very small amounts of material, only, which restricts the analytical deapth of the analysis. Importantly, however, the fraction of common proteins shared between the EPCAM affinity purified sample from Paca44 cells is reduced from 37% to just 24% with Panc1 derived vesicles, when MFGE8 rather than Epcam is used as the anchor protein, supporting our contention, that both anchors may enrich different subsets of extracellular vesicles.

### Comparison with exocharta and vesiclepedia

The proteomic compositions of the purified EVs from the pancreatic cancer cells overlap partially with the data described earlier for EVs derived from other cells [22], but enlarge the knowledge with information about vesicles of pancreatic tumour cell origin. This is apparent when the table of proteins from Paca44 (as well from the Panc1 extracellular vesicle samples) is merged with the entries of the proteins from 2013 in the Vesiclepedia version 1.1 and ExoCarta version 4.1 database [15,22]. Both compilations share more than 600 proteins, but more than 200 Paca44 EV proteins (120 proteins out of the Epcam pure EV proteins) are not included in either database. Furthermore, 150 proteins of affinity purified Panc1 EVs (98 from EPCAM pure and 89 proteins from MFGE8 pure EVs) are still missing from the database (the Additional file 2: Table S1 contains this information in cell type specific files assigned in a separate column). Among these proteins are many SLC proteins and other transmembrane glycoproteins like Claudine 12, GPR56 or PVR and related proteins which were described as expressed by cancer cell lines.

In summary, our measurement of EVs provides a more in depth view of the composition of the EVs of pancreatic tumour cells, and will significantly enlarge the databases published thus far.

#### The affinity purification leads to the loss of particular protein complexes

The following results comparatively describe the different samples with respect to the origin and functional classification of the proteins given by the annotations of the gene ontological entries of the cellular compartments.

**Figure 4:**
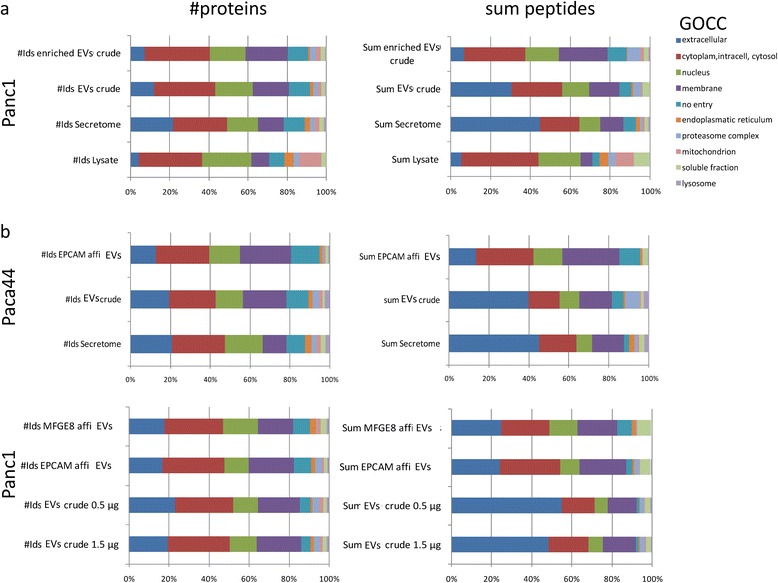
**Top10 gene ontology classification to cellular compartment.** The percentages of the numbers of proteins (left) as well as of the sum of the peptides measured (right) were given. **a)** The crude EV preparations resulted in an increased number of cytoplasmic and membranous originating proteins and a reduction of extracellular proteins, compared to the secretome and lysate of Panc1. The “enriched crude” was named the *in silico* enriched fraction, representing the proteins which were more frequently found in crude EV samples than in the secretomes. (The analysis for HPDE samples were given in Additional file 1: Figure S5). **b)** By the affinity purification procedure, the number of membranous proteins and cytoplasm originating proteins was drastically expanded and the extracellular classified proteins were reduced.

The data show that the compositions of the EV samples differed from the secretome and also from the lysate (Figure 4a). The crude EV samples held about twofold more membrane proteins (near 20%) than the lysate or secretomes. In addition, 25% of the membranous proteins in the EV samples were measured with more than 1.5-fold more peptides than in the secretome (see the display “enriched EV crude” Figure 4a). Otherwise, the mitochondrial proteins found in the lysate were significantly lost in the crude EV samples. Taken together, the crude EV samples represent selective parts of the cellular proteins, not small bin bag*s* of cells. Furthermore, the data indicated that proteins originating from the cytoplasm, nucleus and proteasome complex were additionally enriched in the crude vesicle preparations. The analysis of HPDE samples provided similar results (Additional file 1: Figure S5). In EV preparation from HPDE the number of membranous originated proteins also increased easily but the reduction of extracellular proteins in secretomes is not as effective as those in Paca44 and Panc1. There are some more matrix and structural proteins (fibronectin, laminins, collagens, tubulins), ribonucleoproteins and enzymes in the HPDE EVs.

Based on the GOCC classification, we examined protein groups which were reduced or enriched by the affinity purification. The affinity purified samples of the Paca44 contained more membranous and cytoplasmic proteins than the crude ones (Figure 4b). In contrast, the proportion of extracellular proteins in the affinity pure samples decreased by 8%, and the number of corresponding peptides by 30%. The proteins exclusively measured in the affinity pure samples increased the part of the membrane proteins and also the number of proteins not merged with the GOCC entry. Similar results were obtained for the affinity purification of the Panc1 samples, although to a weaker extent. The proteasome proteins have been efficiently reduced from near 10% of the peptides to less than 1% in the affinity pure samples from both cell lines. This result fits well with the immune detection of the proteasome subunit displayed in Figure 2.

To calculate the amount of “contaminants” in the crude preparation, we analysed the proteins more frequently found in the crude as in the secretome and the affinity pure samples. This could be done after normalization of the mass spectrometry data by the sum of the peptides found in the whole sample (for details, see Materials and Methods). Proteins (#214) lost by the affinity procedure, but frequently measured in the crude samples of the Paca44, were functionally clustered in 4 groups: proteasomal proteins, histones, aminopeptidases and membrane proteins such as semaphorins. Furthermore, we found about 140 proteins in the crude exosomes, which were partially removed or absent in the affinity pure samples, and frequently found in the whole secretome. Proteins like laminins, ribosomal proteins and the giant protocadherin FAT1, as well as the cathepsins and glycosidases, belong to this group.

**Figure 5:**
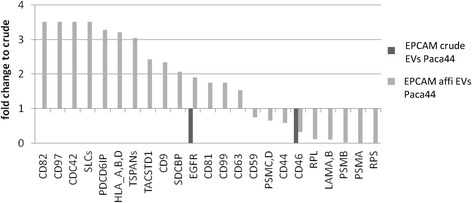
**Affinity purification procedure is accompanied by removal and enhancement of different proteins.** Fold changes were calculated from the normalized and corrected sum of the peptides measured for selected proteins and protein families in Paca44 affinity pure and crude EV samples. The numbers of peptides measured in each sample were corrected and normalized as described in particular in the Materials and Methods. Proteins, e.g. tetraspanins and CD proteins, are enriched, whereas laminins and proteasome proteins were removed.

We observed that some tetraspanins and further membrane proteins were efficiently enriched by affinity purification; however, some membrane proteins like CD44 and CD59, also previously described as exosomal, were reduced through the affinity protocol (Figure 5). The amount of CD9 or TACSTD1/Epcam in the eluates differed according to the efficiency of the procedure, and also with the samples and antibodies used. This suggests the existence of vesicles devoid of TACSTD1/Epcam.

#### Enriched proteins in affinity pure samples: functional implications

##### Clustering by functional analyses

The proteins enriched in the purified samples were filtered by the 1.3-fold change between the normalized and corrected sums of the peptides found from the pure to the crude samples.

**Figure 6:**
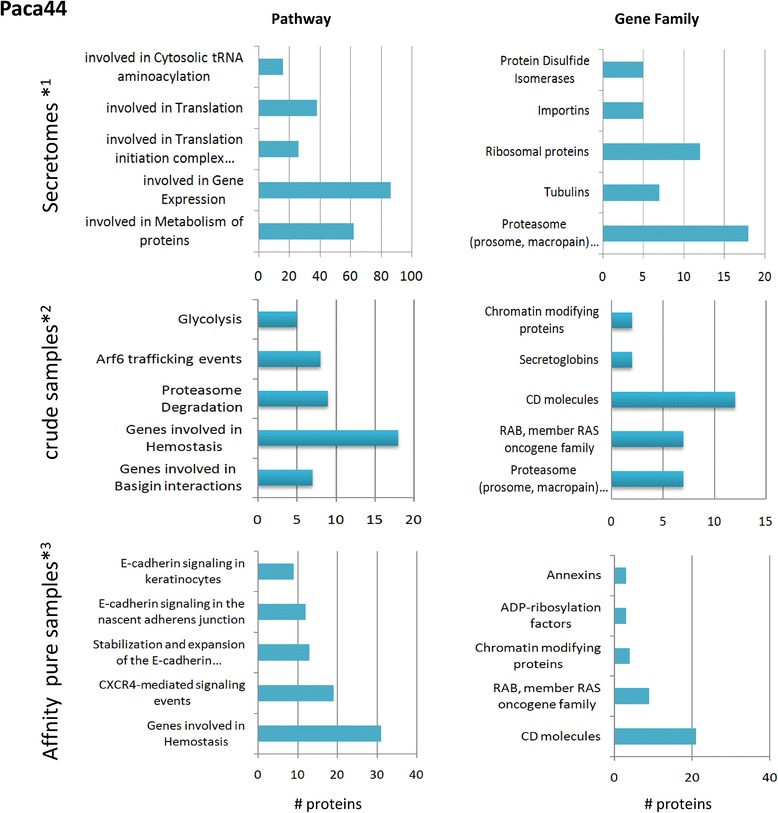
**Comparison of found protein accessions across the Paca44 samples with the ToppGene Suite functional analyses.** The enrichment of the CD molecules, small GTP binding proteins as well as annexins were overrepresented in the affinity pure samples, and the released proteins derived from the ribosomes, proteasomes and tubulins were reduced. *^1^#899 Paca44 proteins were enriched in the secretome, calculated by filtering with fold change (FC) crude EV/secretome < 0.7; *^2^#183 Paca44 proteins were enriched in exosome crude samples filtered by FC crude EV/secretome >4.3; *^3^#342 Paca44 proteins were enriched in affinity purified exosomes filtered by FC affinity pure/crude >1.5 and FC affi pure/secretome > 3; the criteria for analyses are given in Additional file 1: Figure S6.

Focusing on the Paca44 affinity sample, nine functional clusters were built by DAVID clustering from 325 enriched proteins: first group with small GTP-binding proteins (ras family); second group with membrane proteins (CD molecules, tetraspanins, integrins and SLC transporters); third group with endosomal proteins like CHMP4B (function in MVB formation) or VPS37b (responsible for trafficking in MVBs); fourth group with members of the armadillo-repeat containing protein family; fifth group with SH3 domain containing proteins; sixth group with integrin family proteins; seventh group with GTPase activity proteins, like RhoF; eighth group with ATPases; and ninth group with receptor protein kinases like MET, EPHA2 or YES1. More than half of these proteins could not be categorized by DAVID. We reanalysed the data with ToppGene Suite software resulting in a small increase of the number of clustered proteins. The data gave qualitative similar results. A comparative overview across the samples is given in Figure 6 by the Top 5 functional clustering restricted to the gene families and pathway categorizations. Similarities to the Paca44 in clustering were also found for Panc1 samples (and in HPDE restricted to crude EVs), as shown in Additional file 1: Figures S6 a, b. Small GTP binding proteins (ras protein family), annexins, CD molecules and other membranous proteins were enriched by purification. Some differences were in the amount of proteins like histones and some proteasomal proteins in Panc1. This may be due to the different cell types and to the lower purification effectiveness in Panc1 as indicated above.

##### Functional implications for EV-mediated carcinogenic mechanisms

**Figure 7:**
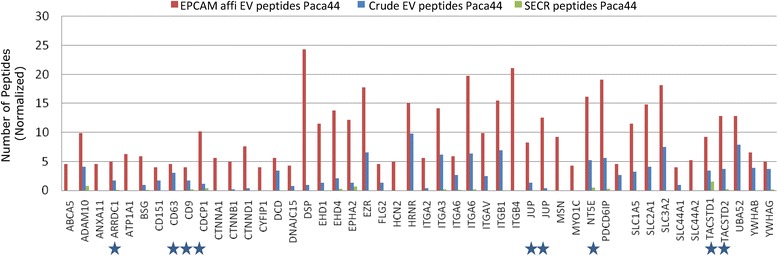
**Top50 proteins enriched in Paca44 affinity pure EV samples in comparison to crude samples and secretomes.** The groups of proteins were selected by fold change FC affinity pure/crude >1.3 and FC affinity pure/secretome > 3, and measured with the minimal 2 peptides. The number of peptides measured in each sample was corrected and normalized as described in the Materials and Methods. The proteins marked with asterisks were tested by specific antibodies in the immune blots (see Additional file 1: Figure S8).

Because of the high efficiency of the affinity purification in Paca44, we focused on the proteins found exclusively and enriched in affinity pure samples of this cell line. The top 50 affinity enriched proteins in Paca44 are shown in Figure 7 (and for Panc1 in Additional file 1: Figure S7). Many membrane associated proteins enriched in the affinity pure samples are known to have modulatory functions in the ECM composition and cell-cell communication. We have selected proteins, which were overrepresented in the samples of the cancer cells, as compared to HPDE as model for normal cells. The proteins Jup/gamma Catenin (CTNNG), CUB domain containing protein (CDCP1), ecto-5′-nucleotidase (CD73/NT5E), radixin (RDX), TACSTD2/Trop2, and arrestin domain containing protein 1 (ARRDC1) were analysed by immune blots. The specific antibodies recognized proteins more efficiently in the affinity pure samples than in the flow through of the affinity columns (Additional file 1: Figure S8).

Of special interest is the protein ARRDC1, which was discussed as a protein responsible for the budding of microvesicles from the plasma membrane [23,24], called ARMMs (ARRDC1-mediated microvesicles). Otherwise, we also found members of the ESCRTIII multiprotein complex, like CHMP4B/VPS32B (function in multivesicular body MVB formation), or ESCRTI complex, like VPS37b (responsible for trafficking in MVBs). The lysosomal proteins Lamp3/CD63 and TSG101, rab7, rab11, rab9, as well as EHDs 1–4 are also compounds of affinity pure samples and are described as endocytotic recycling regulators.

#### Are EV cancer specific proteins suitable as biomarkers and as anchors for vesicle isolation from body fluids? Implications for translational research

The proteomic analysis of the samples should contribute to identify new pancreatic cancer biomarker candidates which are preferentially released by the EVs of pancreatic cancer cells and are suitable in order to use them for the isolation of cancer specific vesicles from patient fluids. An example of proteins enriched in affinity pure EVs of both cancer cell lines was NT5E/CD73, ecto-5′-nucleotidase, which is a plasma membrane protein that catalyses the conversion of extracellular nucleotides to membrane-permeable nucleosides (especially the adenosine release from AMP). As we expected based on the proteomic results, the amount of protein NT5E/CD73 was increased in the affinity pure samples of Paca44, compared to the flow through detected by immune blots (Additional file 1: Figure S8).

The choice of NT5E/CD73 as a potential cancer biomarker was supported by previous findings that three out of five pancreatic cancer cell lines released more peptides of NT5E/CD73 in the secretomes, compared to normal epithelial model cells HPDE (Klein-Scory, S. unpublished data). Additionally, the expression of NT5E/CD73 is upregulated in pancreatic cancer on transcriptional level [25].

**Figure 8:**
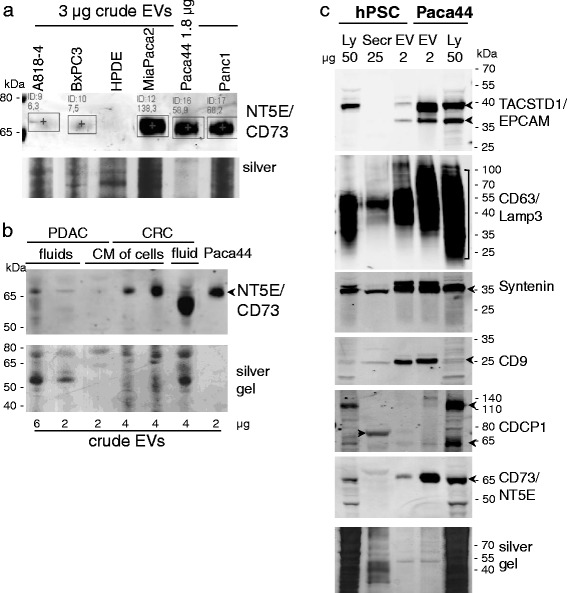
**Release of different amounts of CD73/NT5E proteins: a)** **NT5E/CD73 was detected in EV samples of pancreatic carcinoma cell lines in different amounts.** No NT5E/CD73 was found in the samples of the normal model cells HPDE. The corresponding silver stained gel part is shown for the control. **b)** Ascites derived from PDAC and CRC (colorectal carcinoma) patients and the conditioned medium (CM) of primary cancer cells (isolated from ascites) were used to prepare the EV samples. Samples were tested for NT5E/CD73 by western blot. Additionally, samples of the CRC patients were checked. **c)** Human pancreatic stellate cells (HPSC) also release some of the EV proteins, but in a lesser amount than the pancreatic cancer cell line. An example of corresponding silver stained gel is given. Ly, lysate; Secr, secretome; EV, EV crude preparation.

We tested for this protein in the EV samples of other pancreatic cancer cells and in the ascites fluid of patients. In the EV samples derived from the model cell line HPDE no NT5E/CD73 could be detected, whereas all tested carcinoma cells delivered NT5E/CD73 by vesicle release (Figure 8a). NT5E/CD73 could also be detected in small amounts of EVs from the ascites of PDAC patients (Figure 8b). Are epithelially derived pancreatic cancer cells the exclusive sources of the EV specific proteins found in the ascites of cancer patients?

In order to test whether one of the EV proteins can also be released by other cell types associated with pancreatic cancer, we analysed corresponding samples from human immortalized pancreatic stellate cells hPSC in comparison to those from carcinoma cell lines. The more common EV proteins, like Syntenin/SDCBP and CD9, were detectable in similar amounts in hPSC EVs as in the Paca44 derived ones, but especially the proteins NT5E/CD73 and CDCP1, were increased in the EVs of Paca44, more so than in EVs of hPSCs (Figure 8c). It should be mentioned that hPSCs are immortalized cells which, themselves, are able to form anaplastic tumours when injected into nude mice [26].

The proof of NT5E/CD73 positive vesicles in body fluids suggests that the EV specific proteins might be suitable as anchors for cancer vesicles, but the source of the vesicles is not restricted to epithelial cells.

## Discussion

During early tumour development, the local tissue microenvironment shifts to a more growth promoting state, and tumour promoting mechanisms modulate tissue homeostasis. The components released by tumour cells, as well as by stromal cells, define this state.

Here, we describe the proteomic complexity of the microenvironment of pancreatic tumour cells by analysing the composition of the whole secretome and of the EVs. Particularly the EVs are described as players in the context of tumour stroma interaction [27]. They are containers in which signal molecules are partially protected against proteolysis by stroma components. The functions associated with or described for EVs are manifold [14], and are executed by the vesicular components.

Not only proteins (as we focused on) are delivered by EVs, but nucleic acids (mRNAs, miRNAs and tRNA fragments) are also released together with the EVs [28,29]. It should be noted that it is not clear if the nucleic acids are really a cargo of the vesicles or only associated outside of the vesicles. The process of the potentially selective packaging of different nucleic acids into EVs is still poorly understood. The affinity procedure described herein, together with the deep sequencing analysis of nucleic acids, will provide further knowledge about this subject (Malas, B. unpublished data).

Affinity purification of EVs succeeds with TACSTD1/Epcam and enlarges the knowledge about proteomic composition of pancreatic cancer released EVs. Here, we used this procedure successfully for Paca44 and Panc1 to selectively catch EVs. Our analyses allow the distinction between EVs, proteasomal and ribosomal complexes and the soluble secreted components of pancreatic cancer cells. This is possible through comparison of the deep proteomic description of immune affinity purified samples, to the whole and to fractionated samples of the extracellular proteome.

### Heterogeneity of the vesicles

### Two different origins: oncosomes, ectosomes (ARMMs) and exosomes

EVs share form, density and many characteristic cargos, but are different in their subcellular origins and in their size [5,10]. Some EVs bud (or are shed) directly from the plasma membrane and are called ectosomes, microvesicles or ARMMs. The so called oncosomes or tumour derived microvesicles, TMV, have a broader range of sizes and are often described as a mixed population of microvesicles sharing features described for exosomes and ectosomes [9]. These interpretations are based on the analyses of EVs prepared by procedures not sufficient to differentiate between vesicle populations. The ARMM release depends on the interaction between ARRDC1 and the tetraspanin TSG101. Exosomes, smaller than 120 nm, in restricted view are produced by inward budding of the endosomal membrane, accumulate as intraluminal vesicles, and are secreted by the fusion of multivesicular bodies (MVB) with the plasma membrane. Syntenin, Alix and ESCRT proteins are involved in the formation and release of EVs from the MVB [30].

The affinity pure vesicles derived from the pancreatic cancer cells as presented in this work have the attributes of both, of plasma membrane origin (ectosomes) and of MVB origin (exosomes).The ectosomal proteins ARRDC1 and tetraspanin TSG101 are present in affinity pure vesicle preparations from Paca44 cells, and the EVs from Paca44 seem to be a mixture of both vesicle types. In contrast to ARRDC1, the number of proteins originating from the endosomes, lysosomes or MVBs, like charged multivesicular body proteins, are more frequently found in Paca44 EVs. Therefore, we conclude that the affinity pure samples contain more exosomes than ectosomes.

### Cells release different vesicle fractions with cell line specific compositions

The proteomic analyses displayed many common characteristics of the Epcam affinity purified vesicles from both cell lines analysed here, as presented in the results section (loss of proteasomal and ribosomal proteins, loss of ECM proteins, enrichment of membrane proteins etc.). Nevertheless, the overlap in the number of proteins identified appears moderate (in the range of 40%). This may be due, first, to limitations in the procedure, namely, that the small amounts of material available limit the analytical depth. Another important reason is the individual features of the cell line used an aspect that is not surprising in view of the comprehensive differences in their transcriptomic profiles, their origin and their differentiation. The cell line Panc1 displays fewer ductal features and is described as an epitheloid cell line (see ATCC). The low expression level of TACSTD1/Epcam was suggested to indicate more acinar properties of Panc1 cells [19]. In contrast Paca44 is considered as a pure ductal cell line [31]. So, cell line specific components of extracellular vesicles have to be expected.

Another important aspect is the evidence for the existence of diverse types of vesicles released from one cell line. When an alternative protein, namely MFGE8, was used as an anchor for immune capture of vesicles released from Panc1 cells, both samples from Panc1 cells displayed more pronounced differences, for example, in the relative amount of CD9 and TACSTD1/Epcam in the eluates and the flow through fractions as shown by Western blotting. Moreover, when compared to the Epcam sample from Paca44 cells the overlap among the proteins identified in mass spectrometry is further decreased, supporting our contention, that different types of vesicles are released.

Tauro et al. have recently published results from affinity-purification of vesicles released from the colorectal cancer cell line LIM1863 [32]. When we compare our protein lists with those of Tauro et al., we do not only find in common the typical EV marker proteins like tetraspannins and ESCRT family proteins but also the CRC enriched proteins like ADAM10, TACSTD2/Trop2 and some integrin family proteins. Interstingly, Tauro et al. have also compared fractions immunopurified with two different anchor molecules, namely, the colon-specific membrane protein A33 and TACSTD1/Epcam. The samples are described in detail and the results provide strong evidence for the existence of separate types of vesicles.

This brings up the questions of whether, how and when the sorting of EVs is regulated in cancer cells, and how the packaging of vesicles is regulated. These questions remain to be elucidated, but it appears that the ESCRT (endosomal sorting complex required for transport) machinery with the VPS or CHMPs proteins, the ARF-regulated trafficking mechanisms (review in [33]) and RAB proteins [34] play a role in these processes. Both affinity pure samples of Paca44 and Panc1 contain ESCRT proteins as well as ARF and RAB family members.

Taken together, our data could reveal that pancreatic cancer cells release vesicles mainly derived from endosomal compartments, which we can call exosomes, and the exact composition of the vesicles varies and may be regulated by cell specific mechanisms.

### EVs carry functional components to modify cancer environment

The pure preparations of the EVs allow the detailed proteomic comparison of specific vesicular proteins with non-vesicular protein components found in the whole secretome. Both subproteomes contain signals or modulators addressed to neighbouring cells and more distant surrounding. The EVs, as well as the secreted soluble components, can be bound and ingested by cells, but EVs can also inversely absorb soluble components from the environment.

The protein cargo of EVs and secretomes from pancreatic cancer cells suggests functional activities previously shown for other cellular systems. In some publications using rat models, EVs are suggested to establish premetastatic niches in the periphery of the primary tumour [35]. EVs can do this by preparing an attractive microenvironment at which circulating tumour cells find preferred conditions to survive without immune attack [36]. They can modify the ECM and carry signal modulators and components of signalling pathways, sensing the extracellular environment and modulators of immune cells. The findings of our analyses most strongly support the role of EVs in pancreatic tumours as modulators of the extracellular matrix, regulators of cell-cell contacts and cell migration. Many of the proteins frequently found in pure EVs (Figure 7) are components or regulators of the extracellular matrix (ECM), such as ADAMs, CD9, TROP2, CDCP1 and integrins (see above). Another prominent protein, previously known as tumour derived collagenase stimulating factor (TCSF) and described as a modulator of ECM and immune response (BSG/CD147/EMMPRIN [37]) is also a component of the Paca44 EVs. Recently, it has been shown that CDCP1 expression is required for the attachment of colorectal cancer cells to lung endothelial cells. CDCP1 protein is released by the EVs and could play a role in the extravasation of tumour cells to organ specific metastasis [38].

The function of transmitting the signals from cancer cells to the surrounding or distant environment could be performed by protein kinases (CIT, MAP4K4, Yes1, DDR1, WEE1, SRC) and growth factor receptors like MET, which were measured in Paca44 EVs, together with a high number of small G-proteins (ras, rab and ral families).

The high amount of glycoprotein on the membranes of the EVs could serve in the capture of therapeutic substances, so they did not effectively find the target and became inefficient. This aspect was supported by our findings for the EGF receptor in EVs [39]. Furthermore, the multidrug resistant proteins (ABC family) as well as the SLC transporters providing the import and rapid export of substances into the cells were frequent components of the affinity pure vesicles.

One of the proposed associated functions of the EVs was the modulation of the immune responses [40,41]. A group of proteins specially found in affinity pure samples was functionally clustered to regulate CXCR4-mediated signalling, and an immune modulator is the enzyme NT5E/CD73 which catalyses the release of adenosine [42]. The role of NT5E/CD73 was recently summarized as a suppressor of anti cancer immune responses during carcinogenesis [43].

### Implications for translational applications

EVs isolated from various body fluids, including plasma, malignant ascites, urine, amniotic fluid and saliva by different methods, were used for diagnosis [6], but the origin of the EVs is often not clear. The definition of selective anchors and markers is a prerequisite for the development of an efficient method to isolate EVs and biomarkers. Consequently, our aim was to propose anchor proteins to catch EVs deriving from pancreatic cancer cells. Previous studies using the anchor protein TACSTD1/Epcam showed the amplified release of EVs into the body fluids of ovarian or lung cancer patients [44,45]. The low efficiency of purification using the anchor TACSTD1/Epcam for Panc1 showed that TACSTD1/Epcam could not be considered to be a common anchor for PDAC derived vesicles. The isolation of EVs is restricted to TACSTD1/Epcam expressing cells. Furthermore, it is suggested that this protein is partially processed in body fluids [46]. The other anchor protein, MFGE8, expression also varied in a broad range across the PDAC cell lines, therefore, it is not useful to efficiently isolate pancreatic cancer specific EVs from the body fluids of PDAC patients.

The membrane protein CD73/NT5E was initially the focus in defining pancreatic cancer specific markers. Although the pancreatic carcinoma cells express and release more NT5E/CD73 than the model cell line HPDE, the specificity of this protein for carcinoma cells is limited: for example, the immortalized human pancreatic stellate cells hPSC (and mesenchymal cells) release this protein. Additionally, we found this protein in the ascites of CRC patients. This confirmed that NT5E/CD73 is a component of EVs derived from colorectal cancer cells [45]. Furthermore, its expression is up regulated in other cancer types, such as melanoma, breast and colorectal cancer, and its over expression is correlated with poor prognosis in CRC [47]. The analysis of this candidate illustrates that the EV proteins described in the catalogue herein can be measured in primary material, i.e. in body fluids, and thus provides proof of principle for the importance to include EV derived proteins into biomarker discovery approaches.

## Conclusion

In summary, the affinity purification, together with the deep proteomic analyses of EVs, provided many new components not previously described for EVs derived from pancreatic tumour cells. The data herein clarify the composition of EVs from pancreatic cancer cells in contrast to other released proteins, and discuss these proteins in the context of tumour stroma interactions.

## Materials and methods

### Cell culture and ascites fluid collection

The human pancreatic adenocarcinoma cell line, Paca44, was kindly provided by M. Löhr (Heidelberg, Germany); Panc1 and BxPc3, MiaPaca2 and HPSC (human pancreatic stellate cells) were obtained from the American Type Culture Collection (ATCC, Rockville, MD); and A818-4 cells were obtained from our lab (W.S.). The cells were maintained in supplemented Dulbecco’s Modified Eagle’s Medium (DMEM). Human pancreatic ductal epithelial (HPDE) cells were kindly provided by M.S. Tsao (Toronto) and cultured in defined Keratinocyte-SFM (KFSM, Life technology), as described previously [39].

The collection of ascites was done in the German university hospital, Knappschaftskrankenhaus Bochum, during patient therapy. Ascites was obtained from patients diagnosed with pancreatic cancer or colorectal cancer. All patients provided written informed consent for the use of the samples in research, as approved by the ethics board of the medical faculty of the University of Bochum (http://www.ruhr-uni-bochum.de/ethik/download/Deklaration_Helsinki_2008_engl..pdf). Primary carcinoma cells were isolated from the ascites by centrifugation, and the non-carcinoma cells died within one week. The lymphocytes were lost during the first cell culture procedures. The remaining cells were cultured in DMEM containing 10% foetal calf serum and supplements.

### Preparation of secretome and extracellular vesicle fractions

Secretome preparation was performed as previously described [39]. Cells were grown in standard medium until they reached a confluency of approximately 60-70%. The carcinoma cell lines were then washed three times with DMEM and incubated in a serum-free medium with supplements for 16 hours. This protocol did not measurably increase the rate of cell death, as determined by Casy (Schärfe System, Germany). Afterwards, the ice cooled conditioned media was centrifuged (200 g, 10 min) and passed through 0.2 μm pore filters. A protease inhibitor cocktail was added. The secretome samples were concentrated by ultrafiltration (Amicon Ultra 3 K, Millipore, Schwalbach, Germany) and their concentrations determined in a Bradford protein assay.

The crude preparation of the EV fraction samples was performed as described by van Niel et al. with modifications [48]. In brief, the conditioned media cleared from the cells and cell debris as described above were subjected to ultrafiltration (Amicon Ultra 100 K, Millipore, Schwalbach, Germany) and subsequent ultracentrifugation was performed at 120,000 × g for 90 min at 4°C using a T-890 titanium fixed angle rotor (Sorvall, Langenselbold, Germany). The resuspended pellets were called crude EVs and were stored at −80°C. The preparation of the ascites cell derived EVs was carried out as described for the established pancreatic carcinoma cell lines.

### Density gradient centrifugation

A 15 ml OptiPrep gradient (Axis–Shield, Oslo, Norway) of 5 layers was prepared following the protocol of the manufacturer. The freshly prepared ultracentrifugation pellets were suspended in TBS (50 mM TrisHCl, 135 mM NaCl, 2 mM EDTA pH 7.2) and were added to the top of the gradient. After 18 h of centrifugation in the Surespin 630 rotor of a Sorvall centrifuge at 177,000 × g, 14 fractions of 1 ml each were taken from the top. The fractions were subsequently concentrated by ultrafiltration (Amicon Ultra 100 K, Millipore, Schwalbach, Germany) and analysed by western or dot blots.

### Affinity purification of EVs/exosomes

According to the description in [49], ultracentrifugation pellets, called the crude EV preparation, were incubated with 50 μl TACSTD1/EPCAM coated magnetic beads and separated with μMac columns as described by the manufacturer (Miltenyi Biotech Inc., Bergisch Gladbach, Germany). The isolations of Panc1 exosomes were additionally carried out with anti-MFGE8 or anti-CD63 for 1 hour. Anti-rabbit or anti-mouse beads (50 μl) were added. The magnetically labelled samples were separated by Miltenyi μMac columns as described by the manufacturer (Miltenyi Biotech Inc, Bergisch Gladbach, Germany). After three washes, the columns were removed from the magnet, and the magnetically labelled samples were received using PBS or NP40 containing buffers as indicated. The eluates (and the concentrated flow through of the columns) were analysed.

### Immune detection

The samples, as indicated, were separated on NuPAGE gradient gels (4–12%), and the proteins were transferred to a PVDF membrane in a semi-dry blotting procedure. The following primary antibodies were used: Alix (PDCD6IP; Santa Cruz Biotechnology Inc., Santa Cruz, USA), Syntenin (SDCBP; Synaptic Systems, Göttingen, Germany), Lamp3 (CD63; MX-49.129.5 Santa Cruz Biotechnology Inc.,Santa Cruz, USA), CDCP1 (Cell Signaling Technology, Inc., Danvers, USA), CD9 (Sigma-Aldrich Chemie GmbH, Munich, Germany), MFGE8 (lactadherin, R&D Systems, Wiesbaden, Germany), CD73/NT5E (Sigma-Aldrich ChemieGmbH, Munich, Germany), TACSTD1/Epcam (Biomol, Hamburg, Germany), TACSTD2/Trop2(Abcam, Cambridge, UK), radixin (RDX; Abcam, Cambridge, UK) and arristin (ARRDC1; Abcam, Cambridge, UK). The primary antibodies were detected with species-specific secondary antibodies (goat, rabbit or mouse), each conjugated with a fluorescent dye, Alexa 680 (Invitrogen, Darmstadt, Germany), IRDye 800 (Biomol, Hamburg, Germany) or DyLight™ 800 (Thermo Scientific, Bonn, Germany).

For the dot blot analyses, 3 μl of each fraction were spotted on a nitrocellulose membrane (Millipore, Germany). After the spots had dried, the membrane was blocked and incubated with primary antibodies (anti-Syntenin, anti-CD63/lamp3). The values were calculated by integrated intensities measured using the Odyssey 2.1 software program (LI-COR, USA).

### Illustration of the extracellular vesicles by TEM analysis and PKH67 staining

An aliquot (5 μl) of freshly prepared ultracentrifugation pellets was deposited on Formvar coated grids, and left to adsorb for 30 min. The samples were subsequently negatively contrasted with 1% uranylacetate for 5 min. The grids were viewed under TEM. This procedure followed the protocol described by [50]. The PKH67 green fluorescent cell linker kit (Sigma-Alrich Chemie GmbH, Munich, Germany) was used to label the EVs according to the instruction manual and as described in [51].

### Mass spectrometric analysis

Mass spectrometric analyses are described in [39]. The samples from each cell line and the preparation were separated on 1D NuPAGE gradient gels (4-12% acrylamide, Bis-Tris with MES running buffer; Invitrogen, Darmstadt, Germany). Each lane was cut into 2 mm wide slices as displayed in Additional file 1: Figure S9, and the gel slices were subjected to in-gel digestion with trypsin (Promega, Madison, WI). The supernatant from the tryptic digestion and from the four extraction steps was combined, evaporated and dissolved in H_2_O/FA, 99.9/0.1 v/v.

A nanoscale LC-MS analysis of the tryptic peptides was performed using the nanoACQUITYUPLC system (Waters, Eschborn, Germany) which was coupled online to an LTQ Orbitrap XL mass spectrometer (Thermo Scientific, Bremen, Germany). Data were acquired by scan cycles of one FTMS scan with a resolution of 60,000 and a range from 370 to 2,000 m/z, in parallel with six MS/MS scans in the ion trap of the most abundant precursor ions. The MS/MS spectra were searched with the MASCOT search engine (Matrix Science, London, UK; version 2.2) against the human MSIPI database. The peptide mass tolerance for the database searches was set to 5 ppm, and the fragment mass tolerance was set to 0.5 Da. Furthermore, the proteins were considered to be identified if more than one unique peptide had an individual ion score exceeding the MASCOT identity threshold (ion score cut-off of 22–23). Identification under the applied search parameters refers to a False Discovery Rate (FDR) < 3.5% and a match probability of p < 0.05, where p is the probability that the observed match is a random event.

Each slice was analysed separately and MS/MS data were not merged prior to the protein database search to maintain the information about the molecular weight of each protein, peptide matches and identification score. In this way, the protein catalogues from the human pancreatic cell secretomes, lysates, crude extracellular vesicles (exosomes) and affinity pure EVs were assembled.

### *In silico* comparison of proteomics data

Additional file 2 was compiled for all identified proteins using the IPI number, protein score, protein description and the number of identified peptides (peptide matches).

Normalization of the label free mass spectrometry data was performed according to [52]. The number of peptides of a protein in the different samples was corrected using the sum of the measured peptides. For example, for the Paca44 cell line, 22,965 peptides were found in all slices of the secretome, 5,336 peptides in the crude EV sample, and 3,042 peptides in the affinity pure samples of this cell line (Table 1). To compare these with the Paca44 secretome, the number of matches/peptides was normalized using the correction factors between the sum of the measured peptides corrected by a factor of 4.3 for the Paca44 crude and 7.5 for the Paca44 affinity pure samples. The normalizations were done over the samples per cell line.

To describe more deeply the entirety of proteins measured in the affinity pure samples, we analysed the protein lists with two functional classification tools (DAVID software package and ToppGene Suite software). For the DAVID analysis (database version 6, 2009) [53] and for the ToppGene Suite software [54], the IPI numbers of the proteins were used and analysed against whole entries in the database. With the ToppGene Suite software, the Top5 results of the clustering were displayed across the samples.

The comparison of the mass spectrometry data with the Vesiclepedia database was performed with downloadable files from the homepage, Vesiclepedia 1.1 (http://microvesicles.org/download; [22]) filtered for human and aliases of the gene symbols converted by DAVID. The ExoCarta database 4.1 was downloaded from www.exocarta.org [15].

## Additional files

Additional file 1:
**S1-S9 compiled in a single PDF. S1.** Vesicular character of the crude EV preparations: a) Only the treatment with detergent containing buffers led to the passage of EV proteins through a 100 kDa cut off filter. b) The PKH67 green fluorescent cell linker kit (Sigma-Alrich Chemie GmbH, Munich, Germany) was used to label the EVs according to the instruction manual and as described in [54]. Ultracentrifugation pellets were suspended in 100 μl tris based saline with exosome depleted foetal calf serum; 10 μl anti CD326 FITC was added, and incubated 10–30 min at 4°C. By filling up to 6 ml TBS, the samples were washed, then centrifuged at 120,000 g for 1 h. The supernatant was removed completely and the pellets were resuspended in 0.5 ml TBS and analysed with Facs. **S2.** Selection of cell lines and anchor proteins to isolate pure EV fractions. a) Paca44 contained the highest amount of TACSTD1/EPCAM protein, whereas Panc1 showed a significantly lower TACSTD1/EPCAM detection level in the exosomes. The EV preparations were checked for Syntenin and the proteasome subunits (PSU). b) In contrast to the other pancreatic cells, MFGE8ishighly expressed only in Panc1 and detectable also in HPDE in the crude EV preparation, but not sufficient for effective affinity purification of HPDE EVs. **S3.** Control experiments for affinity purification. Epcam proteins are only detectable in eluates, if the inserted sources containedEVs. **S4.** Venn diagram illustrate the comparison of secretomes and EVs Paca44, Panc1 and HPDE samples (a,b ) The crude EV proteins analyses enlarge the number of proteins found by secretome measurements and deepened the view of released proteins by pancreatic cells. The secretomes of the three different cell lines share nearly 50% of proteins. However, the crude EV comparison is influenced by the different number of measured proteins of different samples. The venn diagram of Panc1 affinity pure samplesusing EPCAM or MFGE8 as anchor proteins (c) shows that both affinity pure samples share 173 proteins, which comprise 41% and 59% ofeach sample. **S5.** S5HPDE secretome and crude EV classified to cellular compartment by gene ontology (TOP 10). The percentages of the numbers of proteins (left) as well as of the sum of the peptides measured (right) were given. In EV preparation from HPDE the number of membranous originated proteins also increased easily but the reduction of extracellular proteins in secretomes is not as effective as in Paca44 and Panc1. **S6.** TOP5 ToppGene Suite categorizations: Lists of proteins of samples from Panc1 (a) and from HPDE (b) were subjected to the functional enrichment analysis by ToppGene Suite. Cut offs of the categorization as well as the compilations of the results are given. **S7.** Top 50 proteins enriched in Panc1 affinity pure EV samples in comparison to crude samples and secretomes filtered by FC affi pure/crude EVs > 1.3 and FC affi pure EVs/secretome > 3. The number of peptides measured in each sample was corrected and normalized as described in the Materials and Methods. Marked by stars are proteins also included in the Top 50 affinity pure Paca44 EVs. **S8.** Proteins more frequently measured by mass spectrometry in affinity pure samples of Paca44 were analysed by immunoblot using specific antibodies. **S9.** Images of gels with slices: Protein samples of each cell line were resolved in NuPAGE gradient gels 4-20% (life technologies). After electrophoresis, the gels were stained with an MS compatible Krypton staining procedure according to the manufacturer’s protocol(Thermo Scientific, Bonn, Germany). The gel pictures were drawn using a Licor Odyssee 680 nm scanner. The gels were cut in 29slices and 15 slices as indicated. Each gel piece was separately prepared and analysed by mass spectrometry. Additional file 2:
**Extended tables of mass spec data as an excel file.**

## Abbreviations

CM: Conditioned medium
EV: Extracellular vesicle
ECM: Extracellular matrix
PDAC: Pancreatic ductal adenocarcinoma
