# Supplementary material for: New insights in the composition of extracellular vesicles from pancreatic cancer cells: implications for biomarkers and functions
Source: Proteome Sci. 2014 Nov 18;12:50. doi: 10.1186/s12953-014-0050-5 (PMC4251850; doi:10.1186/s12953-014-0050-5)
Supplement: Additional file 1: — S1-S9 compiled in a single PDF. S1. Vesicular character of the crude EV preparations: a) Only the treatment with detergent containing buffers led to the passage of EV proteins through a 100 kDa cut off filter. b) The PKH67 green fluorescent cell linker kit (Sigma-Alrich Chemie GmbH, Munich, Germany) was used to label the EVs according to the instruction manual and as described in [54]. Ultracentrifugation pellets were suspended in 100 μl tris based saline with exosome depleted foetal calf serum; 10 μl anti CD326 FITC was added, and incubated 10–30 min at 4°C. By filling up to 6 ml TBS, the samples were washed, then centrifuged at 120,000 g for 1 h. The supernatant was removed completely and the pellets were resuspended in 0.5 ml TBS and analysed with Facs. S2. Selection of cell lines and anchor proteins to isolate pure EV fractions. a) Paca44 contained the highest amount of TACSTD1/EPCAM protein, whereas Panc1 showed a significantly lower TACSTD1/EPCAM detection level in the exosomes. The EV preparations were checked for Syntenin and the proteasome subunits (PSU). b) In contrast to the other pancreatic cells, MFGE8ishighly expressed only in Panc1 and detectable also in HPDE in the crude EV preparation, but not sufficient for effective affinity purification of HPDE EVs. S3. Control experiments for affinity purification. Epcam proteins are only detectable in eluates, if the inserted sources containedEVs. S4. Venn diagram illustrate the comparison of secretomes and EVs Paca44, Panc1 and HPDE samples (a,b ) The crude EV proteins analyses enlarge the number of proteins found by secretome measurements and deepened the view of released proteins by pancreatic cells. The secretomes of the three different cell lines share nearly 50% of proteins. However, the crude EV comparison is influenced by the different number of measured proteins of different samples. The venn diagram of Panc1 affinity pure samplesusing EPCAM or MFGE8 as anchor proteins (c) shows that both affinity pure [file 12953_2014_50_MOESM1_ESM.pdf]

Additional file 1 legends

S1. Vesicular character of the crude EV preparations: a) Only the treatment with detergent containing buffers led to the passage of EV proteins through a 100 kDa cut off filter. b) The PKH67 green fluorescent cell linker kit (Sigma-Alrich Chemie GmbH, Munich, Germany) was used to label the EVs according to the instruction manual and as described in [54]. Ultracentrifugation pellets were suspended in 100 µl tris based saline with exosome depleted foetal calf serum; 10 µl anti CD326 FITC was added, and incubated 10–30 min at 4°C. By filling up to 6 ml TBS, the samples were washed, then centrifuged at 120,000 g for 1 h. The supernatant was removed completely and the pellets were resuspended in 0.5 ml TBS and analysed with Facs.

S2. Selection of cell lines and anchor proteins to isolate pure EV fractions. a) Paca44 contained the highest amount of TACSTD1/EPCAM protein, whereas Panc1 showed a significantly lower TACSTD1/EPCAM detection level in the exosomes. The EV preparations were checked for Syntenin and the proteasome subunits (PSU). b) In contrast to the other pancreatic cells, MFGE8 is highly expressed only in Panc1 and detectable also in HPDE in the crude EV preparation, but not sufficient for effective affinity purification of HPDE EVs.

S3. Control experiments for affinity purification. Epcam proteins are only detectable in eluates, if the inserted sources contained EVs.

S4. Venn diagram illustrate the comparison of secretomes and EVs Paca44, Panc1 and HPDE samples (a,b) The crude EV proteins analyses enlarge the number of proteins found by secretome measurements and deepened the view of released proteins by pancreatic cells. The secretomes of the three different cell lines share nearly 50% of proteins. However, the crude EV comparison is influenced by the different number of measured proteins of different samples. The venn diagram of Panc1 affinity pure samples using EPCAM or MFGE8 as anchor proteins (c) shows that both affinity pure samples share 173 proteins, which comprise 41% and 59% of each sample.

S5 HPDE secretome and crude EV classified to cellular compartment by gene ontology (TOP 10). The percentages of the numbers of proteins (left) as well as of the sum of the peptides measured (right) were given. In EV preparation from HPDE the number of membranous originated proteins also increased easily but the reduction of extracellular proteins in secretomes is not as effective as in Paca44 and Panc1.

S6. TOP5 ToppGene Suite categorizations: Lists of proteins of samples from Panc1 (a) and from HPDE (b) were subjected to the functional enrichment analysis by ToppGene Suite. Cut offs of the categorization as well as the compilations of the results are given.

S7. Top 50 proteins enriched in Panc1 affinity pure EV samples in comparison to crude samples and secretomes filtered by FC affi pure/crude EVs > 1.3 and FC affi pure EVs/secretome > 3. The number of peptides measured in each sample was corrected and normalized as described in the Materials and Methods. Marked by stars are proteins also included in the Top 50 affinity pure Paca44 EVs.

S8. Proteins more frequently measured by mass spectrometry in affinity pure samples of Paca44 were analysed by immunoblot using specific antibodies.

S9. Images of gels with slices: Protein samples of each cell line were resolved in NuPAGE gradient gels 4-20% (life technologies). After electrophoresis, the gels were stained with an MS compatible Krypton staining procedure according to the manufacturer’s protocol (Thermo Scientific, Bonn, Germany). The gel pictures were drawn using a Licor Odyssee 680 nm scanner. The gels were cut in 29 slices and 15 slices as indicated. Each gel piece was separately prepared and analysed by mass spectrometry.

Additional figure S1

a

Paca44 crude EVs

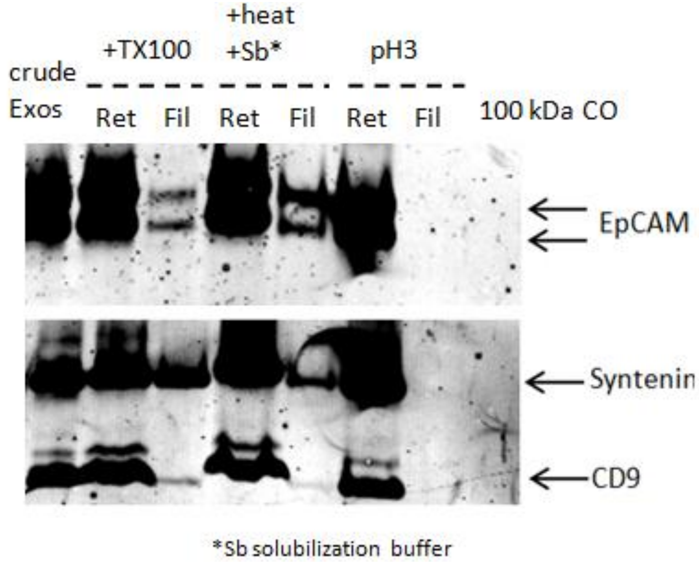

b

Exosomes labeled with PKH67 transfered to recipient cells

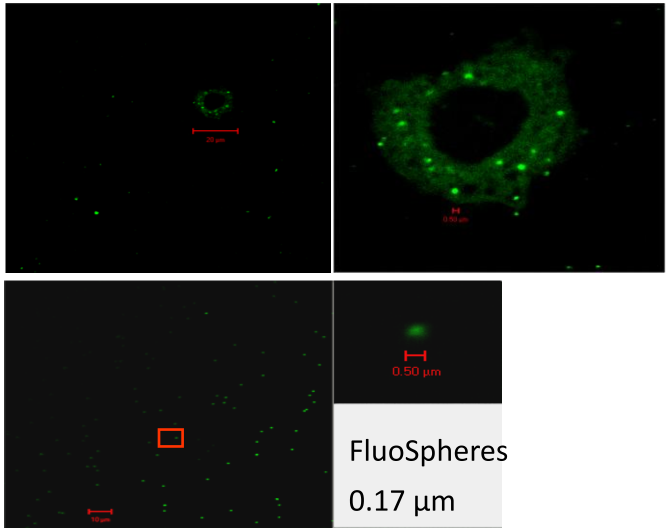

a) Only the treatment with detergent containing buffers leads to the passage of exosomal preoteins through a 100kDa cut off filter.

Crude exosomal preparations were treated with different buffers destroying membrane and the samples were subjected to a 100kDa cut off ultrafiltration. The transmembrane protein TACSTD1/Epcam and CD9 passaged through the filter, if the samples were treated with Triton X100 or treated with a 60°C hot solubilization buffer (containing 50mM Hepes pH7.5, 10% (w/v) Glycerol, 1% Triton X100, 150 mM NaCl, 0.1 mM MgCl2, 50 mM Na_3VO_4, 50 mM NaF, 1 mM PMSF, 10µg/ml aprotinin, 10µg/ml trypsine inhibitor*), whereas the pH decrease could not faciliate the passage of these membraneous proteins.*

b) Membranous specific fluorescent agents PKH67 stain EVs.

Additionally to examine whether the preparation contained vesicles, we labeled the crude preparation with the membranous specific fluorescent agent PKH67 and treated unstained cells with this labeled and filtered ultracentrifuge pellet. As it is expected for membranous vesicles, the exosomal treated cells got spotted fluorescence whereas the cells treated with flow through of PKH67 labeled samples remained dark.

Additional figure S2 Selection of cell line and anchor proteins to isolate pure exosomal fractions.

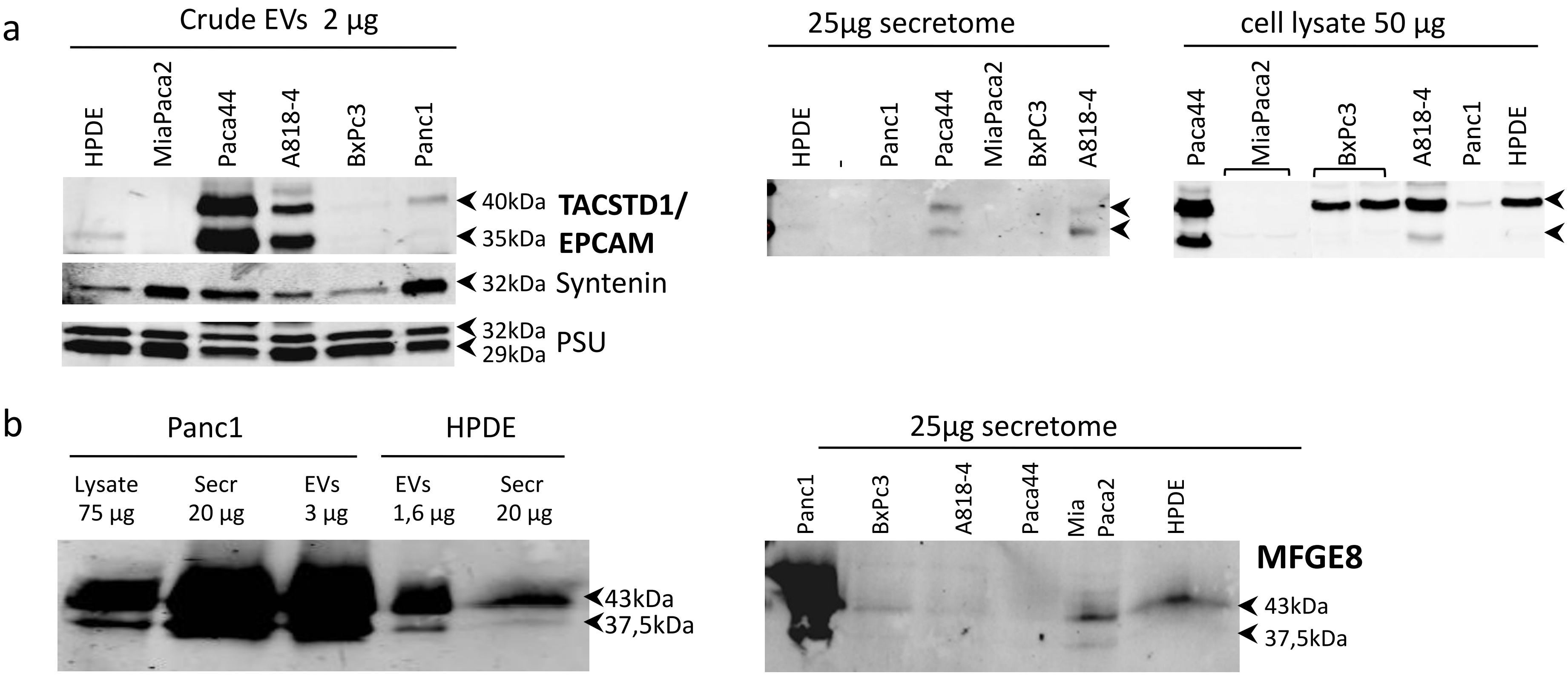

Additional figure S3 Control experiments to affinity purification.

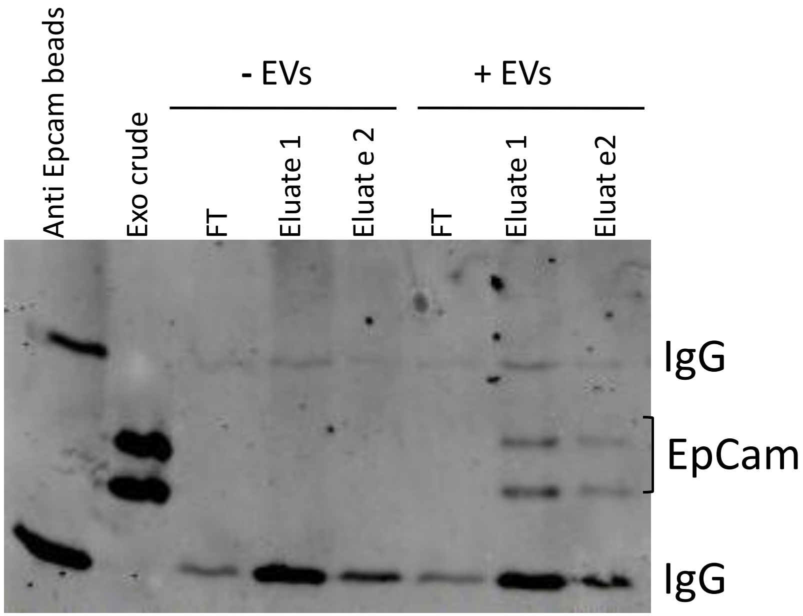

additional figure S4a Summerized proteomics data in venn diagrams

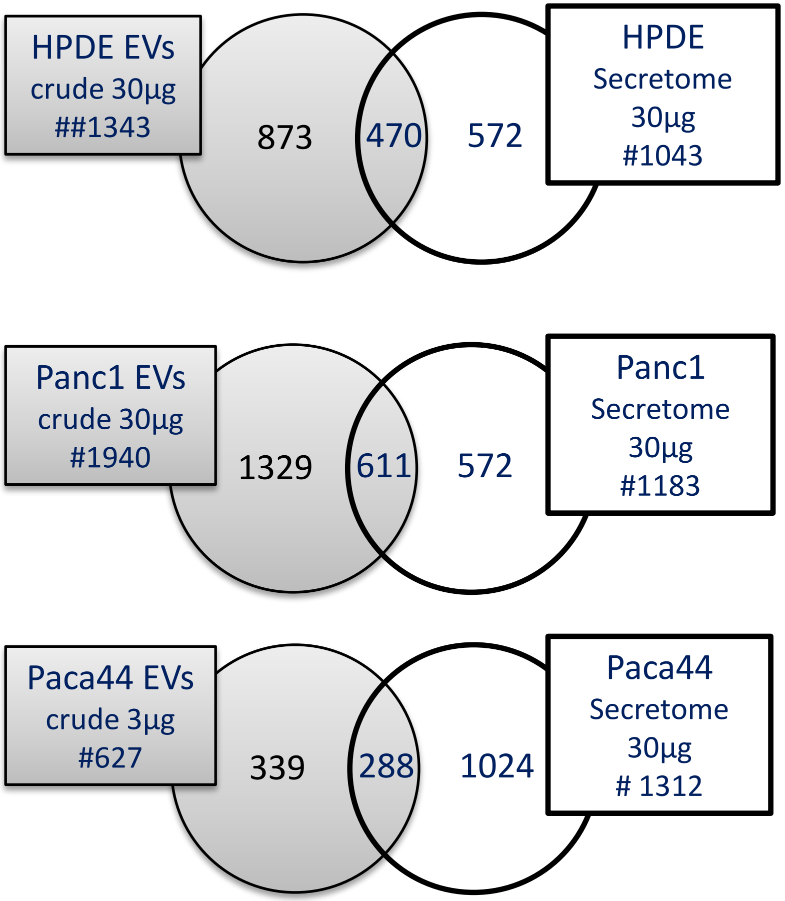

additional figure S4 b

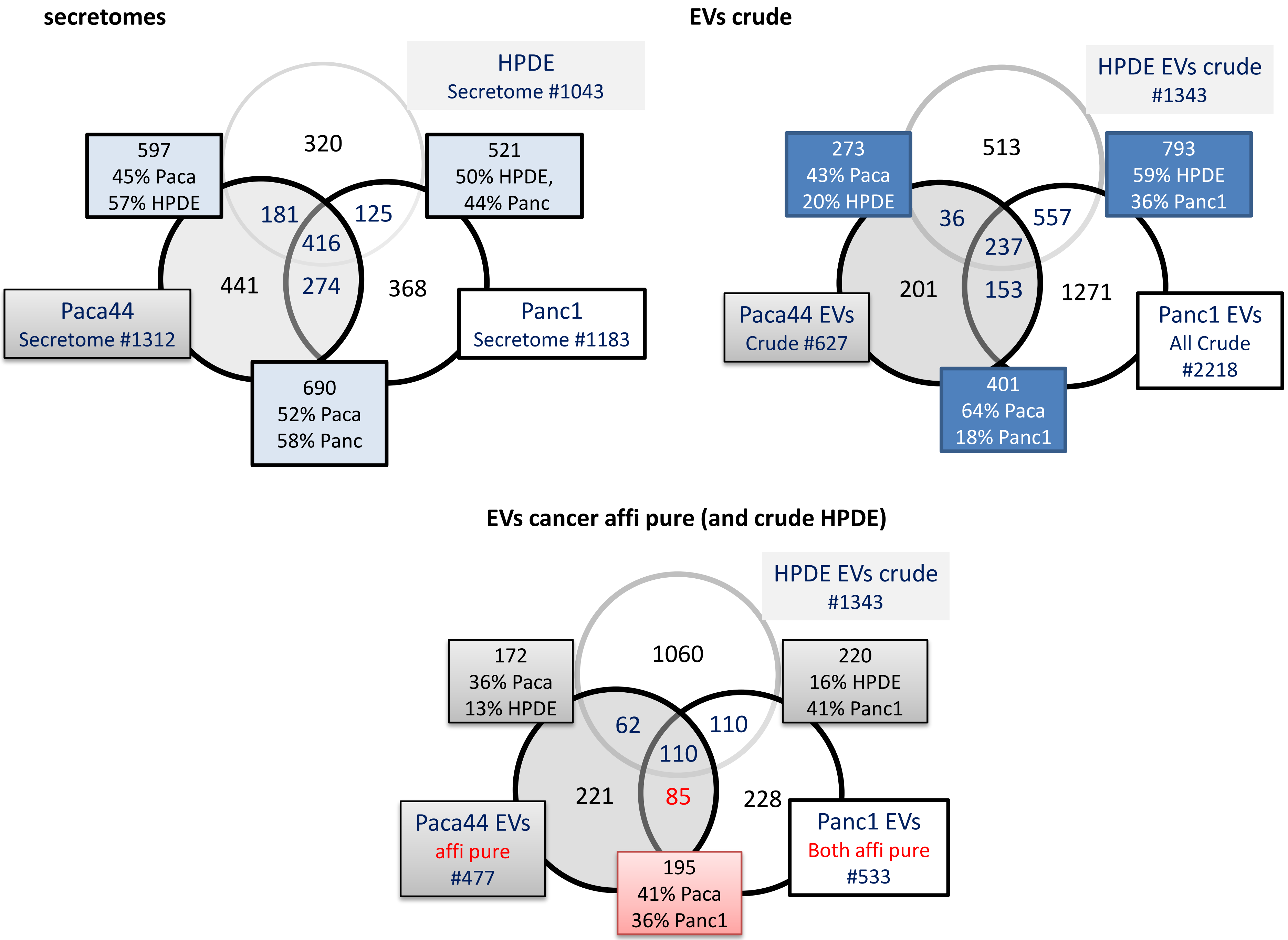

Additional figure S4c

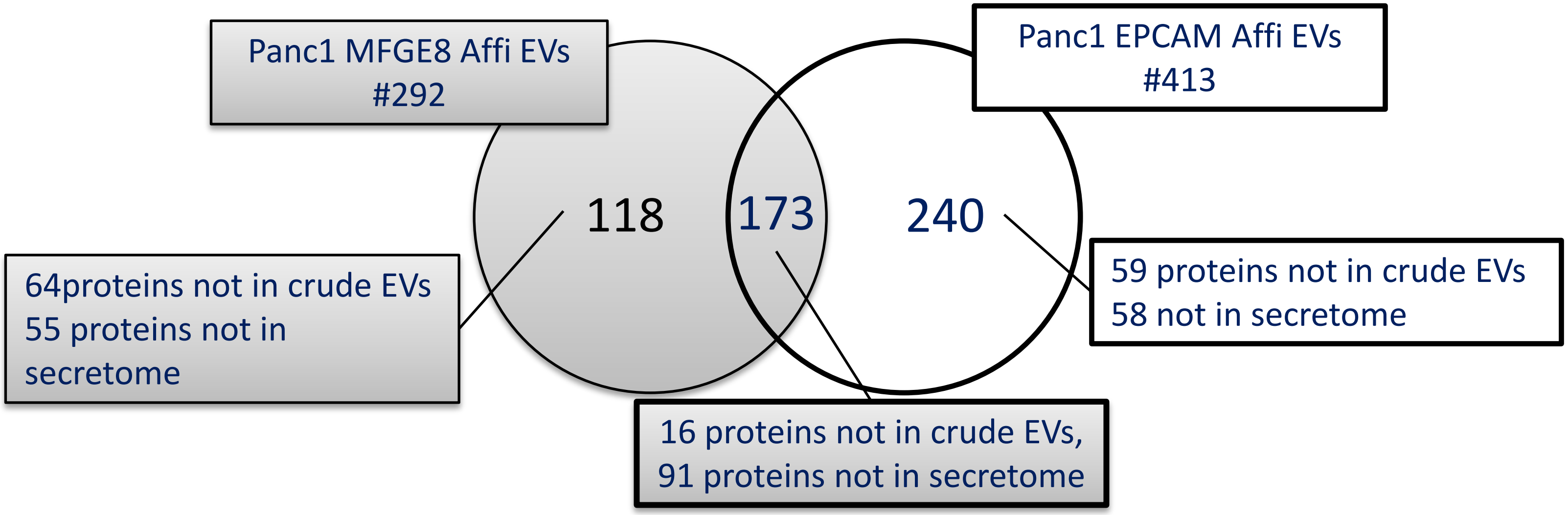

additional figure S5

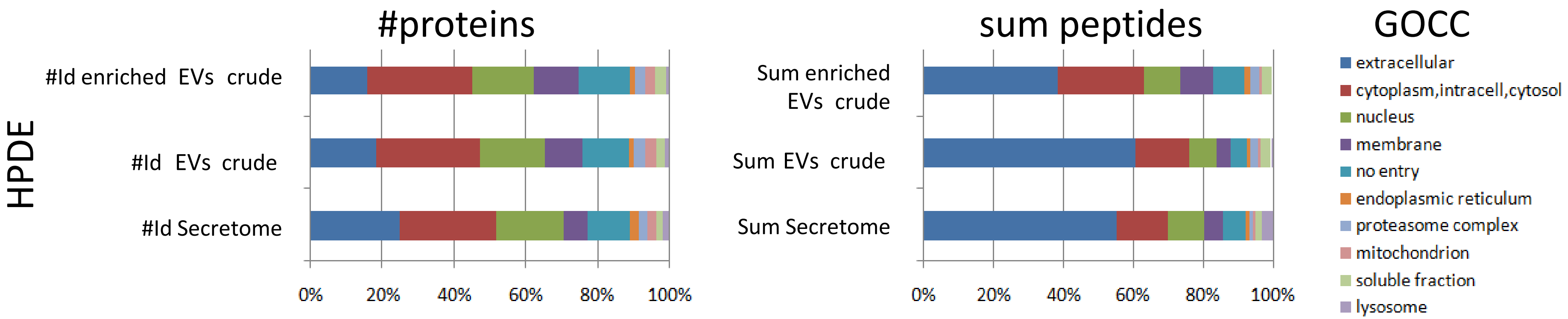

Additional figure S6a TOP5 ToppGene Suite categorizations of Panc1

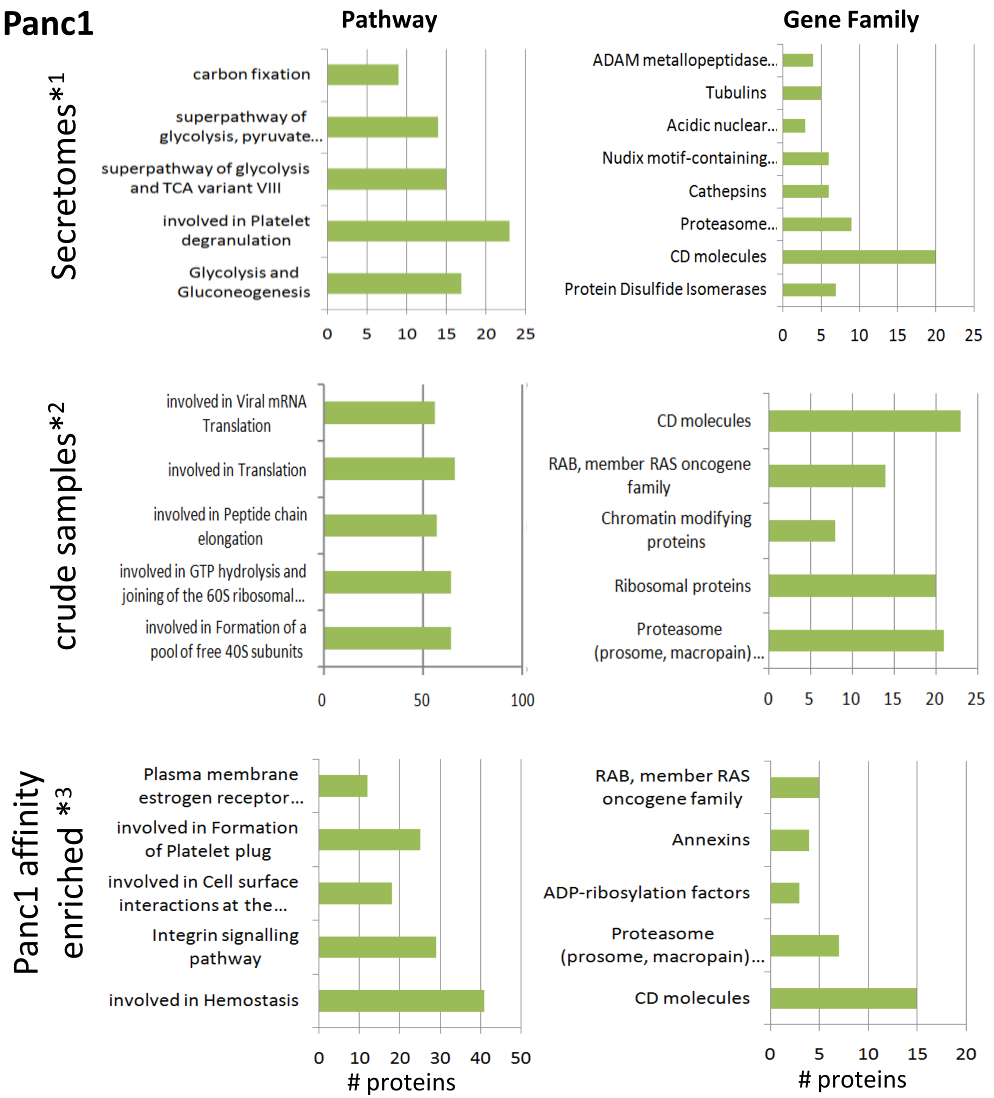

Correction and Cutoff:				
category	Correction	Cutoff	Min	Max
GO: Molecular Function	Bonferroni	0.05	1	1500
GO: Biological Process	Bonferroni	0.05	1	1500
GO: Cellular Component	Bonferroni	0.05	1	1500
Domain	Bonferroni	0.05	1	1500
Pathway	Bonferroni	0.05	1	1500
Interaction	Bonferroni	0.05	1	1500
Gene Family	Bonferroni	0.05	1	1500
Random sampling size in analysis:	0			
Minimun feature count in test set:	2			

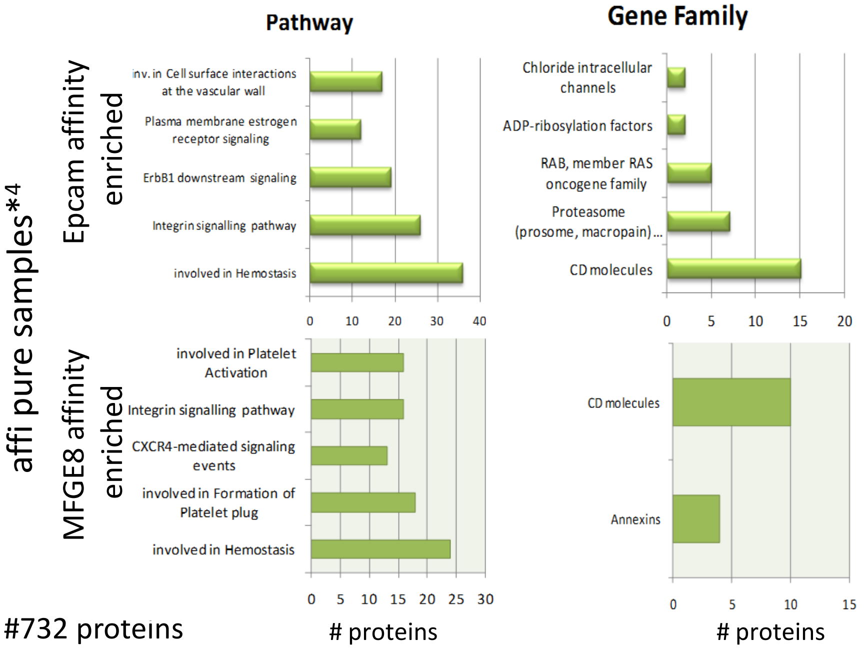

*[1]**Panc1:** Enriched in secretome, filtered by FC crude EVs/secretome < 0.7; #732 proteins

*[2]**Panc1**: Enriched in EVs crude, filtered by FC crude EVs/secretome >8.8 (avarage of FC)

*[3]**Panc1**: affi enriched FC affi pure EVs/crude EVs >1,5 ; EPCAM affi or MFGE8 affi pure EVs #456 proteins

*[4]**Panc1**: affi enriched FC EPCAM affi pure Evs /crude EVs >1,5 ; #356; FC MFGE8 affi pure EVs/crude EVs >1,5; #241 proteins

additional figure S6b

HPDE

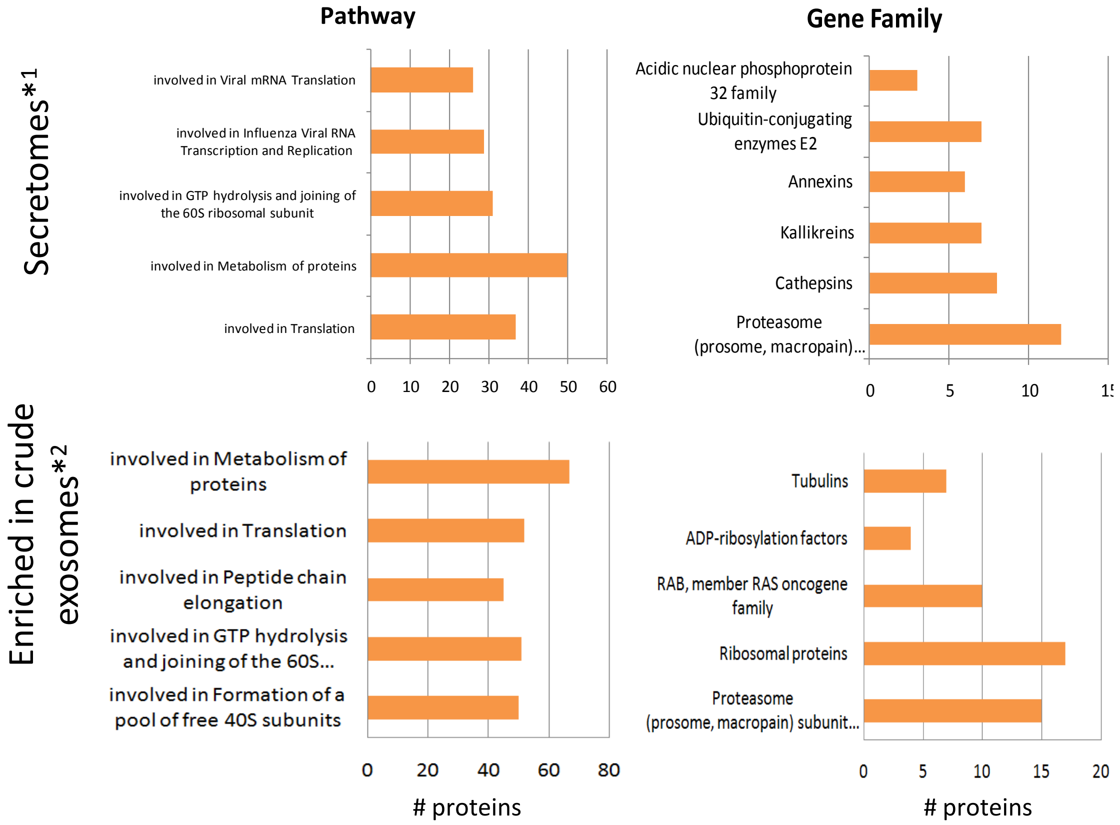

Correction and Cutoff:				
category	Correction	Cutoff	Min	Max
GO: Molecular Function	Bonferroni	0.05	1	1500
GO: Biological Process	Bonferroni	0.05	1	1500
GO: Cellular Component	Bonferroni	0.05	1	1500
Domain	Bonferroni	0.05	1	1500
Pathway	Bonferroni	0.05	1	1500
Interaction	Bonferroni	0.05	1	1500
Gene Family	Bonferroni	0.05	1	1500
Random sampling size in analysis:	0			
Minimun feature count in test set:	2			

*[1]Secretome enriched FC crude EVs/secretome < 0.7: #833 proteins in HPDE

*[2]Enriched in crude EVs: 512 analysed from 585 filtered by FC crude EVs/secretome>7.4 more than average

Additional figure S7 Top 50 proteins enriched in Panc1 affinity pure exosomal samples

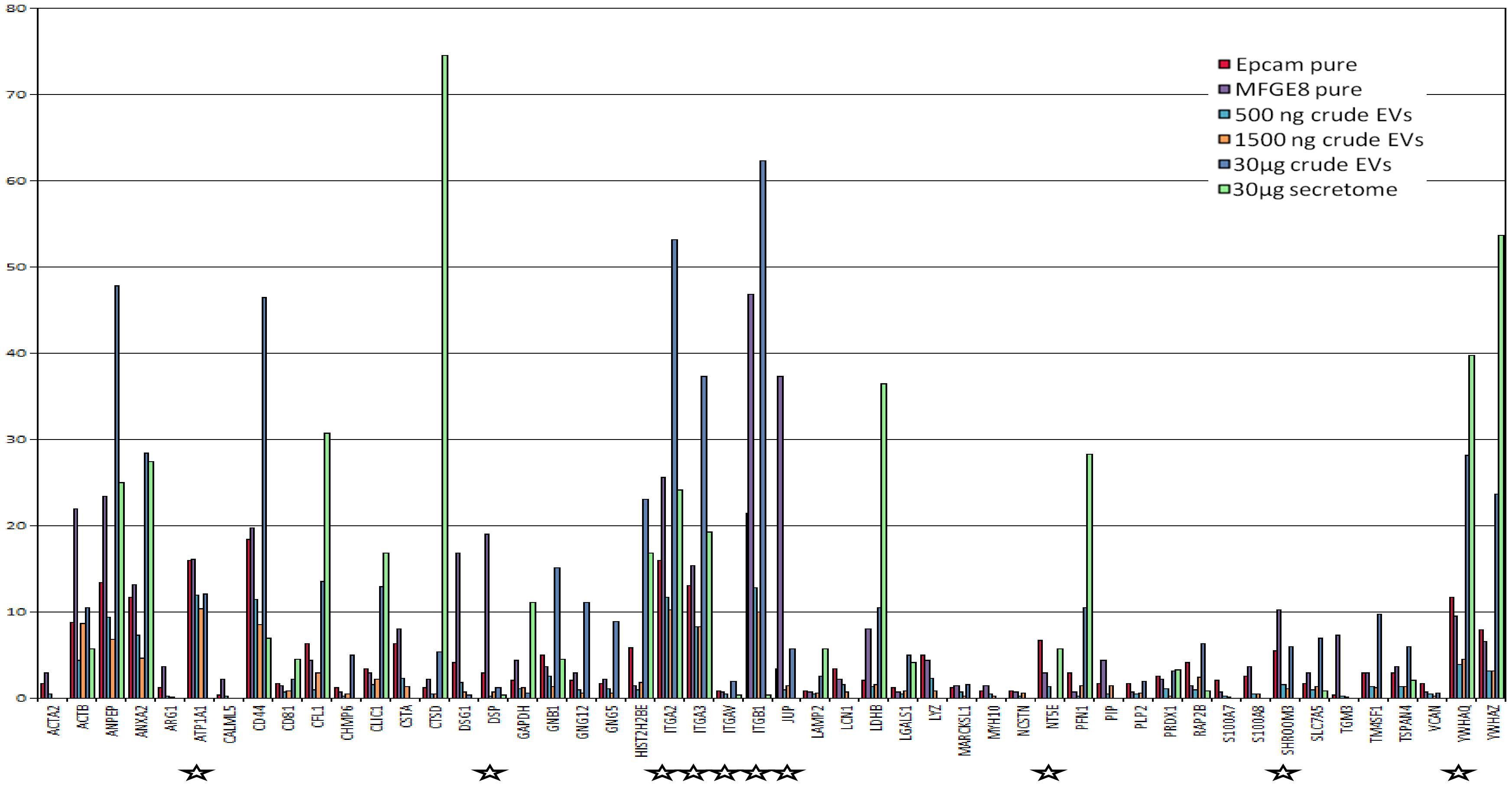

☆ Also included in TOP50 of affinity pure Paca44 EVs

Additional figure S8

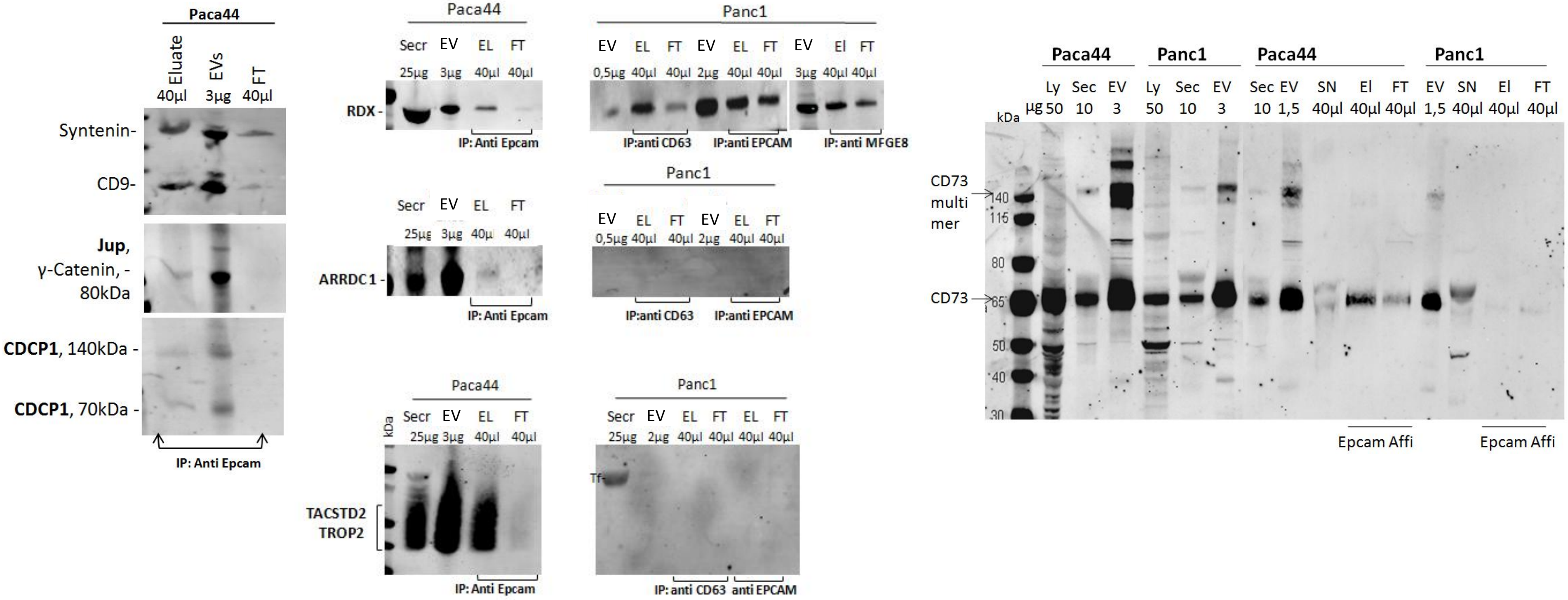

Additional figure S9

30µg secretome Paca44

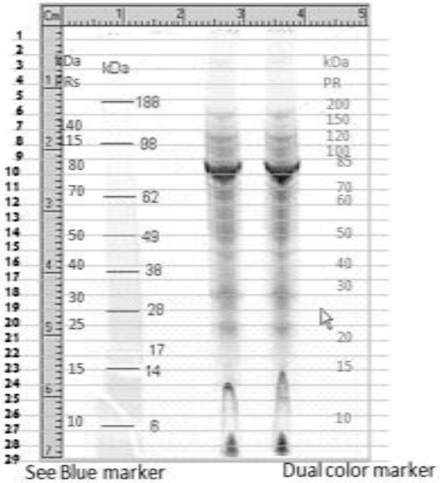

30µg secretome

HPDE Panc1

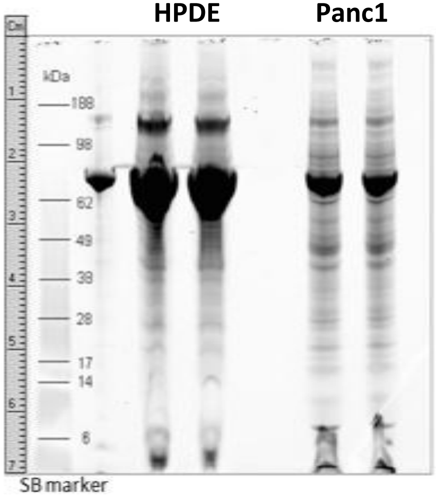

30 µg extracellular vesicles

HPDE Panc1

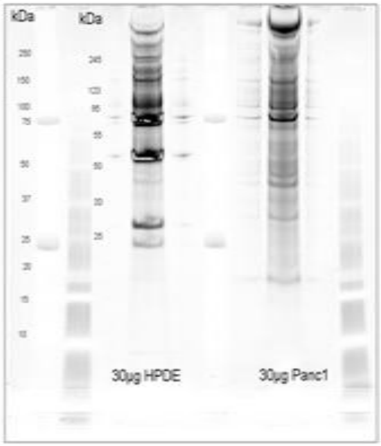

30µg cell lysate

Panc1

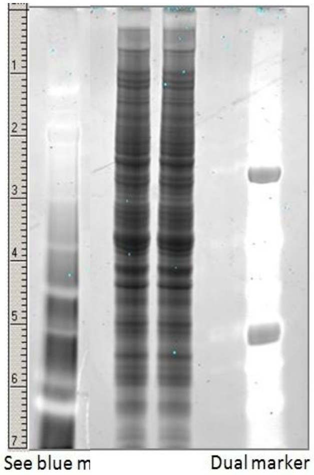

Paca44 extracellular vesicles

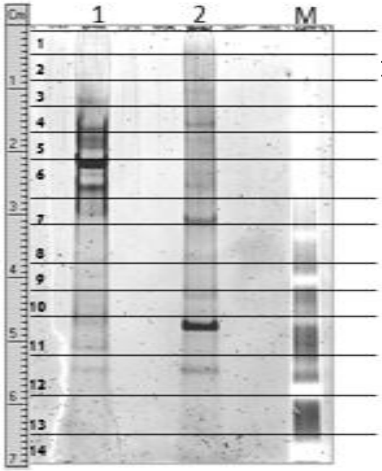

1. 3 µg Paca44 Evs crude
2. from 9µg Evs IP Eluate anti EPCAM

Panc1 extracellular vesicles

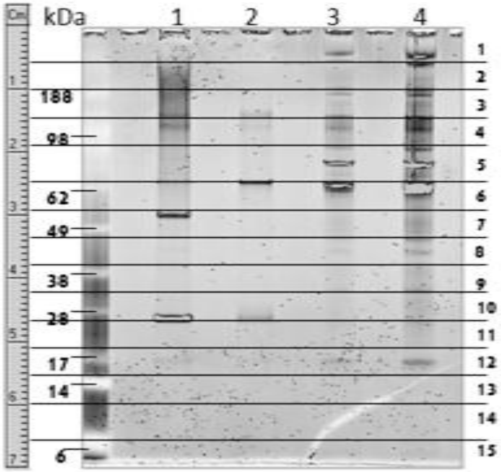

1. from 15 µg Panc1 EVs, IP Eluate; anti EPCAM coated beads3 µg Paca44 EVs crude
2. From 15 µg Panc1 Evs, IPEluate; anti MFGE8 + anti mouse beads
3. 300 ng Panc1 EVs crude
4. 1500 ng Panc1 EVs crude
